# Optimal convergence for adaptive IGA boundary element methods for weakly-singular integral equations

**DOI:** 10.1007/s00211-016-0836-8

**Published:** 2016-08-11

**Authors:** Michael Feischl, Gregor Gantner, Alexander Haberl, Dirk Praetorius

**Affiliations:** 1 The Red Centre, School of Mathematics and Statistics, University of New South Wales, Sydney, NSW 2052 Australia; 2 Institute for Analysis and Scientific Computing, TU Wien, Wiedner Hauptstraße 8-10, 1040 Vienna, Austria

**Keywords:** 65D07, 65N38, 65N50, 65Y20

## Abstract

In a recent work (Feischl et al. in Eng Anal Bound Elem 62:141–153, 2016), we analyzed a weighted-residual error estimator for isogeometric boundary element methods in 2D and proposed an adaptive algorithm which steers the local mesh-refinement of the underlying partition as well as the multiplicity of the knots. In the present work, we give a mathematical proof that this algorithm leads to convergence even with optimal algebraic rates. Technical contributions include a novel mesh-size function which also monitors the knot multiplicity as well as inverse estimates for NURBS in fractional-order Sobolev norms.

## Introduction

### Isogeometric analysis

The central idea of isogeometric analysis (IGA) is to use the same ansatz functions for the discretization of the partial differential equation at hand, as are used for the representation of the problem geometry. Usually, the problem geometry ${\varOmega }$ is represented in CAD by means of non-uniform rational B-splines (NURBS), T-splines, or hierarchical splines. This concept, originally invented in [32] for finite element methods (IGAFEM) has proved very fruitful in applications; see also the monograph [9].

Since CAD directly provides a parametrization of the boundary $\partial {\varOmega }$, this makes the boundary element method (BEM) the most attractive numerical scheme, if applicable (i.e., provided that the fundamental solution of the differential operator is explicitly known). However, compared to the IGAFEM literature, only little is found for isogeometric BEM (IGABEM). The latter has first been considered for 2D BEM in [36] and for 3D BEM in [40]. Unlike standard BEM with piecewise polynomials which is well-studied in the literature, cf. the monographs [39, 41] and the references therein, the numerical analysis of IGABEM is widely open. We refer to [35, 37, 38, 42] for numerical experiments, to [44] for fast IGABEM with ${\mathcal {H}}$-matrices, and to [31] for some quadrature analysis. To the best of our knowledge, a posteriori error estimation for IGABEM, however, has only been considered for simple 2D model problems in the recent own works [24, 25]. The present work extends the techniques from standard BEM to non-polynomial ansatz functions. The remarkable flexibility of the IGA ansatz functions to manipulate their smoothness properties motivates the development of a new adaptive algorithm which does not only automatically adapt the mesh-width, but also the continuity of the IGA ansatz function to exploit the additional freedoms and the full potential of IGA. This is the first algorithm which simultaneously steers the resolution and the smoothness of the ansatz functions, and, it may thus be a first step to a full *hpk*-adaptive algorithm.

For standard BEM with discontinuous piecewise polynomials, a posteriori error estimation and adaptive mesh-refinement are well understood. We refer to [1, 11, 12] for weighted-residual error estimators and to [19, 22] for recent overviews on available a posteriori error estimation strategies. Moreover, optimal convergence of mesh-refining adaptive algorithms has recently been proved for polyhedral boundaries [20, 21, 26] as well as smooth boundaries [27]. The work [2] allows to transfer these results to piecewise smooth boundaries; see also the discussion in the review article [8].

While this work focusses on adaptive IGABEM, adaptive IGAFEM is considered, e.g., in [16, 43]. A rigorous error and convergence analysis in the frame of adaptive IGAFEM is first found in [5] which proves linear convergence for some adaptive IGAFEM with hierarchical splines for the Poisson equation, and optimal rates are announced for some future work.

### Model problem

We develop and analyze an adaptive algorithm for the following model problem: Let ${\varOmega }\subset \mathbb {R}^2$ be a Lipschitz domain with $\mathrm{diam}({\varOmega })<1$ and ${\varGamma }\subseteq \partial {\varOmega }$ be a compact, piecewise smooth part of its boundary with finitely many connected components. We consider the weakly-singular boundary integral equation

1.1 $$\begin{aligned} V\phi (x):=-\frac{1}{2\pi }\int _{\varGamma }\log |x-y|\,\phi (y)\,dy = f(x) \quad \text {for all }x\in {\varGamma }_{}, \end{aligned}$$

where the right-hand side *f* is given and the density $\phi$ is sought. We note that (1.1) for ${\varGamma }=\partial {\varOmega }$ is equivalent to the Laplace–Dirichlet problem

1.2 $$\begin{aligned} -{\varDelta } u=0\text { in }{\varOmega }\quad \text {with } u=f\text { on }{\varGamma },\quad \text {where }u:=V\phi . \end{aligned}$$

To approximate $\phi$, we employ a Galerkin boundary element method (BEM) with ansatz spaces consisting of *p*-th order NURBS. The convergence order for uniform partitions of ${\varGamma }$ is usually suboptimal, since the unknown density $\phi$ may exhibit singularities, which are stronger than the singularities in the geometry. In [24], we analyzed a weighted-residual error estimator and proposed an adaptive algorithm which uses this a posteriori error information to steer the *h*-refinement of the underlying partition as well as the local smoothness of the NURBS across the nodes of the adaptively refined partitions. It reflects the fact that it is a priori unknown, where the singular and smooth parts of the density $\phi$ are located and where approximation by nonsmooth resp. smooth functions is required. In [24], we observed experimentally that the proposed algorithm detects singularities and possible jumps of $\phi$ and leads to optimal convergence behavior. In particular, we observed that the proposed adaptive strategy is also superior to adaptive BEM with discontinuous piecewise polynomials in the sense that our adaptive NURBS discretization requires less degrees of freedom to reach a prescribed accuracy.

### Contributions

We prove that the adaptive algorithm from [24] is rate optimal in the sense of [8]: Let $\mu _\ell$ be the weighted-residual error estimator in the $\ell$-th step of the adaptive algorithm. First, the adaptive algorithm leads to linear convergence of the error estimator, i.e., $\mu _{\ell +n} \le Cq^n\mu _\ell$ for all $\ell ,n\in \mathbb {N}_0$ and some independent constants $C>0$ and $0<q<1$. Moreover, for sufficiently small marking parameters, i.e. aggressive adaptive refinement, the estimator decays even with the optimal algebraic convergence rate. Here, the important innovation is that the adaptive algorithm does not only steer the local refinement of the underlying partition (as is the case in the available literature, e.g., [8, 20, 21, 26, 27]), but also the multiplicity of the knots. In particular, the present work is the first available optimality result for adaptive algorithms in the frame of isogeometric analysis. Additionally, we can prove at least plain convergence if the adaptive algorithm is driven by the Faermann estimator $\eta _\ell$ analyzed in [25] instead of the weighted-residual estimator $\mu _\ell$, which generalizes a corresponding result for standard adaptive BEM [23].

Technical contributions of general interest include a novel mesh-size function $h\in L^\infty ({\varGamma })$ which is locally equivalent to the element length (i.e., $h|_T \simeq \mathrm{length}(T)$ for all elements *T*), but also accounts for the knot multiplicity. Moreover, for $0<\sigma <1$, we prove a local inverse estimate $\Vert h^{\sigma }{\varPsi }\Vert _{L^2({\varGamma })} \le C\,\Vert {\varPsi }\Vert _{\widetilde{H}^{-\sigma }({\varGamma })}$ for NURBS on locally refined meshes. Similar estimates for piecewise polynomials are shown in [15, 29, 30], while [3] considers NURBS but integer-order Sobolev norms only.

Throughout, all results apply for piecewise smooth parametrizations $\gamma$ of ${\varGamma }$ and discrete NURBS spaces. In particular, the analysis thus covers the NURBS ansatz used for IGABEM, where the same ansatz functions are used for the discretization of the integral equation and for the resolution of the problem geometry, as well as spline spaces and even piecewise polynomials on the piecewise smooth boundary ${\varGamma }$ which can be understood as special NURBS.

### Outline

The remainder of this work is organized as follows: Sect. 2 fixes the notation and provides the necessary preliminaries. This includes, e.g., the involved Sobolev spaces (Sect. 2.2), the functional analytic setting of the weakly-singular integral equation (Sect. 2.3), the assumptions on the parametrization of the boundary ${\varGamma }$ (Sect. 2.4), the discretization of the boundary (Sect. 2.5), the mesh-refinement strategy (Sect. 2.6), B-splines and NURBS (Sect. 2.7), and the IGABEM ansatz spaces (Sect. 2.8). Section 3 states our adaptive algorithm (Algorithm 3.1) from [24] and formulates the main theorems on linear convergence with optimal rates for the weighted-residual estimator $\mu _\ell$ (Theorem 3.2) and on plain convergence for the Faermann estimator $\eta _\ell$ (Theorem 3.4). The linear convergence for the $\mu _\ell$-driven algorithm is proved in Sect. 4. The proof requires an inverse estimate for NURBS in a fractional-order Sobolev norm (Proposition 4.1) as well as a novel mesh-size function for B-spline and NURBS discretizations (Proposition 4.2) which might be of independent interest. The proof of optimal convergence behaviour is given in Sect. 5. In Sect. 6, we show convergence for the $\eta _\ell$-driven algorithm.

For the empirical verification of the optimal convergence behavior of Algorithm 3.1 for $\mu _\ell$- as well as $\eta _\ell$-driven adaptivity and a comparison of IGABEM and standard BEM with discontinuous piecewise polynomials, we refer to the numerous numerical experiments in our preceding work [24].

## Preliminaries

### General notation

Throughout, $|\cdot |$ denotes the absolute value of scalars, the Euclidean norm of vectors in $\mathbb {R}^2$, the measure of a set in $\mathbb {R}$ (e.g., the length of an interval), or the arclength of a curve in $\mathbb {R}^{2}$. The respective meaning will be clear from the context. We write $A\lesssim B$ to abbreviate $A\le cB$ with some generic constant $c>0$ which is clear from the context. Moreover, $A\simeq B$ abbreviates $A\lesssim B\lesssim A$. Throughout, mesh-related quantities have the same index, e.g., $\mathcal {N}_\star$ is the set of nodes of the partition $\mathcal {T}_\star$, and $h_\star$ is the corresponding local mesh-width etc. The analogous notation is used for partitions $\mathcal {T}_+$ resp. $\mathcal {T}_\ell$ etc.

### Sobolev spaces

For any measurable subset ${{\varGamma }_0}\subseteq {\varGamma }$, let $L^2({{\varGamma }_0})$ denote the Lebesgue space of all square integrable functions which is associated with the norm $\Vert u\Vert _{L^2({{\varGamma }_0})}^2:=\int _{{\varGamma }_0} |u(x)|^2\,dx$. We define for any $0<\sigma \le 1$ the Hilbert space

2.1 $$\begin{aligned} H^{\sigma }({{\varGamma }_0}) := \big \{u\in L^2({{\varGamma }_0})\,:\,\Vert u\Vert _{H^{\sigma }({{\varGamma }_0})}<\infty \big \}, \end{aligned}$$

associated with the Sobolev–Slobodeckij norm

2.2 $$\begin{aligned} \Vert u\Vert _{H^{\sigma }({{\varGamma }_0})}^2 := \Vert u\Vert _{L^2({{\varGamma }_0})}^2 + |u|_{H^{\sigma }({{\varGamma }_0})}^2, \end{aligned}$$

with

2.3 $$\begin{aligned} |u|_{H^{\sigma }({{\varGamma }_0})}^2 := {\left\{ \begin{array}{ll} \int _{{\varGamma }_0}\int _{{\varGamma }_0}\frac{|u(x)-u(y)|^2}{|x-y|^{1+2\sigma }}\,dy\,dx, &{} \text {for }0<\sigma <1\\ \Vert \partial _{\varGamma } u\Vert _{L^2({\varGamma }_0)},&{}\text {for }\sigma =1,\end{array}\right. } \end{aligned}$$

where $\partial _\gamma$ denotes the arclength derivative. For finite intervals $I\subseteq \mathbb {R}$, we use analogous definitions. By $\widetilde{H}^{-\sigma }({{\varGamma }_0})$, we denote the dual space of $H^{\sigma }({{\varGamma }_0})$, where duality is understood with respect to the extended $L^2({{\varGamma }_0})$-scalar product, i.e.,

2.4 $$\begin{aligned} \langle u\,;\,\phi \rangle _{{\varGamma }_0} = \int _{{\varGamma }_0} u(x)\phi (x)\,dx \quad \text {for all }u\in H^{\sigma }({{\varGamma }_0}) \text { and }\phi \in L^2({{\varGamma }_0}). \end{aligned}$$

We note that $H^{\sigma }({\varGamma })\subset L^2({\varGamma })\subset \widetilde{H}^{-\sigma }({\varGamma })$ form a Gelfand triple and all inclusions are dense and compact. Amongst other equivalent definitions of $H^{\sigma }({{\varGamma }_0})$ are for example interpolation techniques. All these definitions provide the same space of functions but different norms, where norm equivalence constants depend only on ${{\varGamma }_0}$; see, e.g., the monographs [33, 34] and the references therein. Throughout our proofs, we shall use the Sobolev–Slobodeckij norm (2.2), since it is numerically computable.

### Weakly-singular integral equation

It is known [33, 34] that the weakly-singular integral operator $V:\widetilde{H}^{-1/2}({\varGamma })\rightarrow H^{1/2}({\varGamma })$ from (1.1) is a symmetric and elliptic isomorphism if $\mathrm{diam}({\varOmega })<1$ which can always be achieved by scaling. For a given right-hand side $f\in H^{1/2}({\varGamma })$, the strong form (1.1) is thus equivalently stated by

2.5 $$\begin{aligned} \langle V\phi \,;\,\psi \rangle _{{\varGamma }} = \langle f\,;\,\psi \rangle _{{\varGamma }} \quad \text {for all }\psi \in \widetilde{H}^{-1/2}({\varGamma }_{}), \end{aligned}$$

and the left-hand side defines an equivalent scalar product on $\widetilde{H}^{-1/2}({\varGamma })$. In particular, the Lax–Milgram lemma proves existence and uniqueness of the solution $\phi \in \widetilde{H}^{-1/2}({\varGamma })$. Additionally, $V:L^2({\varGamma })\rightarrow H^1({\varGamma })$ is well-defined, linear, and continuous.

In the Galerkin boundary element method, the test space $\widetilde{H}^{-1/2}({\varGamma }_{})$ is replaced by some discrete subspace $\mathcal {X}_{\star }\subset {L^{2}({\varGamma }_{})}\subset \widetilde{H}^{-1/2}({\varGamma }_{})$. Again, the Lax–Milgram lemma guarantees existence and uniqueness of the solution ${\varPhi }_\star \in \mathcal {X}_\star$ of the discrete variational formulation

2.6 $$\begin{aligned} \langle V{\varPhi }_\star \,;\,{\varPsi }_\star \rangle _{{\varGamma }} = \langle f\,;\,{\varPsi }_\star \rangle _{{\varGamma }} \quad \text {for all }{\varPsi }_\star \in \mathcal {X}_\star . \end{aligned}$$

Below, we shall assume that $\mathcal {X}_\star$ is linked to a partition $\mathcal {T}_\star$ of ${\varGamma }$ into a set of connected segments.

### Boundary parametrization

Let ${\varGamma } = \bigcup _i{\varGamma }_i$ be decomposed into its finitely many connected components ${\varGamma }_i$. Since the ${\varGamma }_i$ are compact and piecewise smooth as well, it holds

$$\begin{aligned} \Vert u\Vert ^2_{H^{1/2}({\varGamma })} = \sum _{i}\Vert u\Vert ^2_{H^{1/2}({\varGamma }_i)} + \sum _{\begin{array}{c} {i,j} \\ {i\ne j} \end{array}} \int _{{\varGamma }_i}\int _{{\varGamma }_j}\frac{|u(x)-u(y)|^2}{|x-y|^2}\,dy\,dx \simeq \sum _{i} \Vert u\Vert ^2_{H^{1/2}({\varGamma }_i)}; \end{aligned}$$

see, e.g., [25, Section 2.2]. The usual piecewise polynomial and NURBS basis functions have connected support and are hence supported by some *single* ${\varGamma }_i$ each. Without loss of generality and for the ease of presentation, we may therefore assume throughout that ${\varGamma }$ is connected. All results of this work remain valid for non-connected ${\varGamma }$.

We assume that either ${\varGamma }=\partial {\varOmega }$ is parametrized by a closed continuous and piecewise two times continuously differentiable path $\gamma :[a,b]\rightarrow {\varGamma }$ such that the restriction $\gamma |_{[a,b)}$ is even bijective, or that ${\varGamma }\subsetneqq \partial {\varOmega }$ is parametrized by a bijective continuous and piecewise two times continuously differentiable path $\gamma :[a,b]\rightarrow {\varGamma }$. In the first case, we speak of *closed* ${\varGamma }=\partial {\varOmega }$, whereas the second case is referred to as *open* ${\varGamma }\subsetneqq \partial {\varOmega }$.

For closed ${\varGamma }=\partial {\varOmega }$, we denote the $(b-a)$-periodic extension to $\mathbb {R}$ also by $\gamma$. For the left and right derivative of $\gamma$, we assume that $\gamma ^{\prime _\ell }(t)\ne 0$ for $t\in (a,b]$ and $\gamma ^{\prime _r}(t)\ne 0$ for $t\in [a,b)$. Moreover we assume that $\gamma ^{\prime _\ell }(t) +c\gamma ^{\prime _r}(t)\ne 0$ for all $c>0$ and $t\in [a,b]$ resp. $t\in (a,b)$. Finally, let $\gamma _L:[0,L]\rightarrow {\varGamma }$ denote the arclength parametrization, i.e., $|\gamma _L^{\prime _\ell }(t)| = 1 = |\gamma _L^{\prime _r}(t)|$, and its periodic extension. Elementary differential geometry yields bi-Lipschitz continuity

2.7 $$\begin{aligned} C_{{\varGamma }}^{-1} \le \frac{|\gamma _L(s)-\gamma _L(t)|}{|s-t|}\le C_{{\varGamma }}\quad \text {for }s,t\in \mathbb {R}, {\text { with } {\left\{ \begin{array}{ll} |s-t|\le \frac{3}{4}\,L, \text { for closed }{\varGamma },\\ s\ne t\in [0,L], \text { for open }{\varGamma }, \end{array}\right. }}\nonumber \\ \end{aligned}$$

where $C_{{\varGamma }}>0$ depends only on ${\varGamma }$. A proof is given in [28, Lemma 2.1] for closed ${\varGamma }=\partial {\varOmega }$. For open ${\varGamma }\subsetneqq \partial {\varOmega }$, the proof is even simpler.

Let $I\subseteq [a,b]$. If ${\varGamma }=\partial {\varOmega }$ is closed and $|I|\le \frac{3}{4} L$ resp. if ${\varGamma }\subsetneqq \partial {\varOmega }$ is open, the bi-Lipschitz continuity (2.7) implies

2.8 $$\begin{aligned} C_{{\varGamma }}^{-1}|u\circ \gamma _{L}|_{H^{1/2}(I)}\le |u|_{H^{1/2}(\gamma _L(I))}\le C_{{\varGamma }}|u\circ \gamma _{L}|_{H^{1/2}(I)}. \end{aligned}$$

### Boundary discretization

In the following, we describe the different quantities which define the discretization.

**Nodes** $\varvec{z_j=\gamma (\check{z}_j)\in \mathcal {N}_\star }.$ Let $\mathcal {N}_\star :=\big \{z_j\,:\,j=1,\dots ,n\big \}$ and $z_0:=z_n$ for closed ${\varGamma }=\partial {\varOmega }$ resp. $\mathcal {N}_\star :=\big \{z_j\,:\,j=0,\dots ,n\big \}$ for open ${\varGamma }\subsetneqq \partial {\varOmega }$ be a set of nodes. We suppose that $z_j=\gamma (\check{z}_j)$ for some $\check{z}_j\in [a,b]$ with $a=\check{z}_0<\check{z}_1<\check{z}_2<\dots <\check{z}_n=b$ such that $\gamma |_{[\check{z}_{j-1},\check{z}_j]}\in C^2([\check{z}_{j-1},\check{z}_j])$.

**Multiplicity** $\varvec{\#z_j}$ **and knots** $\varvec{\mathcal {K}_\star },\varvec{\check{\mathcal {K}}_\star }.$ Let $p\in \mathbb {N}_0$ be some fixed polynomial order. Each node $z_j$ has a multiplicity $\#z_j\in \{1,2\dots , p+1\}$ with $\#{z}_{0}=\#z_{n}=p+1$. This induces knots

2.9 $$\begin{aligned} \mathcal {K}_\star =(\underbrace{z_k,\dots ,z_k}_{\#z_k\text {-times}},\dots , \underbrace{z_n,\dots ,z_n}_{\#z_n\text {-times}}), \end{aligned}$$

with $k=1$ resp. $k=0$ and corresponding knots $\check{\mathcal {K}}_\star :=\gamma |_{(a,b]}^{-1}(\mathcal {K}_\star )$ resp. $\check{\mathcal {K}}_\star :=\gamma ^{-1}(\mathcal {K}_\star )$ on the parameter domain [*a*, *b*].

**Elements, partition** $\varvec{\mathcal {T}_\star },$ **and** $\varvec{[T]},$ $\varvec{[\mathcal {T}_\star }].$ Let $\mathcal {T}_\star =\{T_1,\dots ,T_n\}$ be a partition of ${\varGamma }$ into compact and connected segments $T_j=\gamma (\check{T}_j)$ with $\check{T}_j=[\check{z}_{j-1},\check{z}_j]$. We define

2.10 $$\begin{aligned}{}[\mathcal {T}_\star ]:=\big \{[T]\,:\,T\in \mathcal {T}_\star \big \} \quad \text {with } [T]:=(T,\#z_{T,1},\#z_{T,2}), \end{aligned}$$

where $z_{T,1}=z_{j-1}$ and $z_{T,2}=z_j$ are the two nodes of $T=T_j$.

**Local mesh-sizes** $\varvec{h_{\star ,T}},$ $\varvec{\check{h}_{\star ,T}}$ **and** $\varvec{h_\star },$ $\varvec{\check{h}_\star }.$ The arclength of each element $T\in \mathcal {T}_\star$ is denoted by $h_{\star ,T}$. We define the local mesh-width function $h_\star \in L^\infty ({\varGamma })$ by $h_\star |_T=h_{\star ,T}$. Additionally, we define for each element $T\in \mathcal {T}_\star$ its length $\check{h}_{\star ,T}:=|\gamma ^{-1}(T)|$ with respect to the parameter domain [*a*, *b*]. This gives rise to $\check{h}_\star \in L^\infty ({\varGamma })$ with $\check{h}_\star |_T=\check{h}_{\star ,T}$. Note that the lengths $h_{\star ,T}$ and $\check{h}_{\star ,T}$ of an element *T* are equivalent, where the equivalence constants depend only on $\gamma$.

**Local mesh-ratios** $\varvec{\check{\kappa }_\star }.$ We define the local mesh-ratio by

2.11 $$\begin{aligned} \check{\kappa }_\star&:=\max \big \{\check{h}_{\star ,T}/\check{h}_{\star ,T'}\,:\,{T},{T}'\in \mathcal {T}_\star \text { with } T\cap T'\ne \emptyset \big \}. \end{aligned}$$

**Fig. 1:**
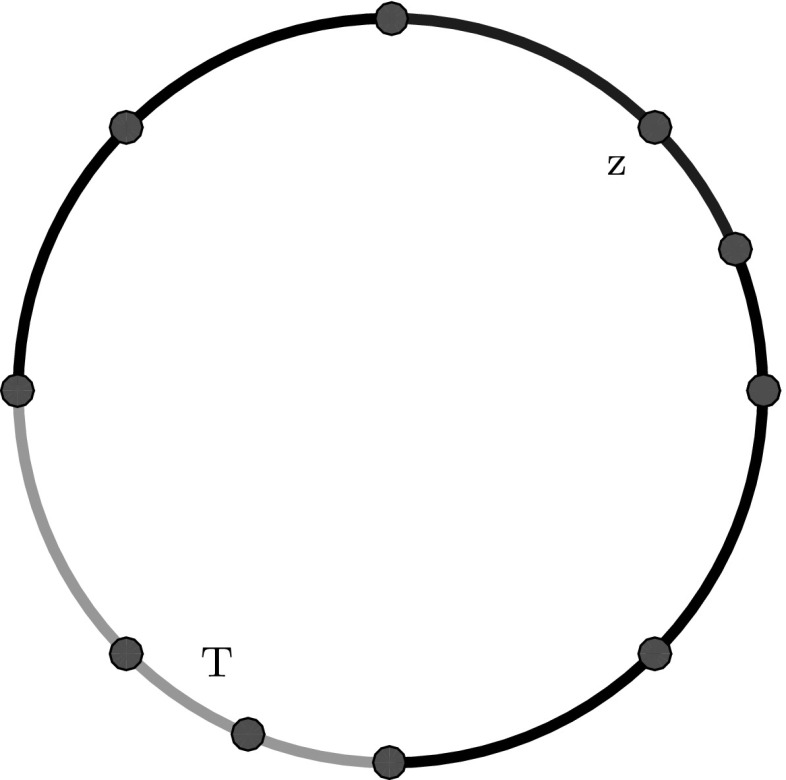
The patch $\omega _\star (z)$ of some node $z\in \mathcal {N}_\star$ resp. the patch $\omega _\star (T)$ are illustrated in *blue* resp. *green*

**Patches** $\varvec{\omega _\star (z)},$ $\varvec{\omega _\star (U)},$ $\varvec{\omega _\star ({\mathcal {U}})},$ **and** $\varvec{\bigcup \mathcal U}.$ For each set $U\subseteq {\varGamma }$, we inductively define for $m\in \mathbb {N}_0$ (Fig. 1)

$$\begin{aligned} \omega _\star ^m(U) := {\left\{ \begin{array}{ll} U\quad &{}\text {if }m=0,\\ \omega _\star (U):= \bigcup \big \{T\in \mathcal {T}_\star \,:\,T\cap U\ne \emptyset \big \}\quad &{}\text {if }m=1,\\ \omega _\star (\omega _\star ^{m-1}(U)) \quad &{}\text {if }m>1. \end{array}\right. } \end{aligned}$$

For nodes $z\in {\varGamma }$, we abbreviate $\omega _\star (z)=:\omega _\star (\{z\}).$ For each set ${\mathcal {U}}\subseteq [\mathcal {T}_\star ]$, we define

$$\begin{aligned} \bigcup \mathcal U := \bigcup \big \{T\in \mathcal {T}_\star \,:\,[T]\in \mathcal U\big \}, \end{aligned}$$

and

$$\begin{aligned} \omega _\star ^m({\mathcal {U}}) := \omega _\star ^m\left( \bigcup {\mathcal {U}}\right) . \end{aligned}$$

### Mesh-refinement

Suppose that we are given a deterministic mesh-refinement strategy $\mathtt{ref}(\cdot )$ such that, for each mesh $[\mathcal {T}_{\star }]$ and an arbitrary set of marked nodes $\mathcal M_\star \subseteq \mathcal {N}_\star$, the application $[\mathcal {T}_{+}] := \mathtt{ref}([\mathcal {T}_\star ],\mathcal M_\star )$ provides a mesh in the sense of Sect. 2.5 such that, first, the marked nodes belong to the union of the refined elements, i.e., $\mathcal M_\star \subset \bigcup ([\mathcal {T}_\star ]{\setminus } [\mathcal {T}_{+}])$, and, second, the knots $\mathcal {K}_\star$ form a subsequence of the knots $\mathcal {K}_{+}$. The latter implies the estimate

2.12 $$\begin{aligned} |[\mathcal {T}_\star ]{\setminus } [\mathcal {T}_{+}]|\le 2(|\mathcal {K}_{+}|-|\mathcal {K}_\star |), \end{aligned}$$

since $[\mathcal {T}_\star ]{\setminus }[\mathcal {T}_+]$ is the set of all elements in which a new knot is inserted and one new knot can be inserted in at most 2 elements of the old mesh, i.e., at the intersection of 2 elements.

We write $[\mathcal {T}_{+}]\in \mathtt{ref}([\mathcal {T}_\star ])$, if there exist finitely many meshes $[\mathcal {T}_1],\dots ,[\mathcal {T}_\ell ]$ and subsets $\mathcal M_{j}\subseteq \mathcal {N}_{j}$ of the corresponding nodes such that $[\mathcal {T}_\star ] = [\mathcal {T}_1]$, $[\mathcal {T}_{+}]=[\mathcal {T}_\ell ]$, and $[\mathcal {T}_{j}] = \mathtt{ref}([\mathcal {T}_{j-1}],\mathcal M_{j-1})$ for all $j=2,\dots ,\ell$, where we formally allow $m=1$, i.e., $[\mathcal {T}_\star ]=[\mathcal {T}_{1}]\in \mathtt{ref}([\mathcal {T}_\star ])$.

For the proof of our main result, we need the following assumptions on $\mathtt{ref}(\cdot )$.

#### Assumption 2.1

For an arbitrary initial mesh $[\mathcal {T}_0]$ and $[\mathbb {T}]:=\mathtt{ref}([\mathcal {T}_0])$, we assume that the mesh-refinement strategy satisfies the properties (M1)–(M3):

1. There exists a constant $\check{\kappa }_{\max }\ge 1$ such that the local mesh-ratios (2.11) are uniformly bounded
  2.13 $$\begin{aligned} \check{\kappa }_\star \le \check{\kappa }_{\max }\quad \text {for all }[\mathcal {T}_\star ]\in [\mathbb {T}]. \end{aligned}$$
2. For all $[\mathcal {T}_\star ],[\mathcal {T}_{+}]\in [\mathbb {T}]$, there is a common refinement $[\mathcal {T}_\star \oplus \mathcal {T}_{+}]\in \mathtt{ref}([\mathcal {T}_\star ]){\cap {\mathtt{ref}}}([\mathcal {T}_{+}])$ such that the knots $\mathcal {K}_\star \oplus \mathcal {K}_{+}$ of $[\mathcal {T}_\star \oplus \mathcal {T}_{+}]$ satisfy the overlay estimate
  2.14 $$\begin{aligned} |\mathcal {K}_\star \oplus \mathcal {K}_{+}|\le |\mathcal {K}_\star |+|\mathcal {K}_{+}|-|\mathcal {K}_0|. \end{aligned}$$
3. Each sequence $[\mathcal {T}_\ell ]\in [\mathbb {T}]$ of meshes generated by successive mesh-refinement, i.e., $[\mathcal {T}_j] = \mathtt{ref}([\mathcal {T}_{j-1}],\mathcal M_{j-1})$ for all $j \in \mathbb {N}$ and arbitrary $\mathcal M_j \subseteq \mathcal {N}_j,$ satisfies
  2.15 $$\begin{aligned} |\mathcal {K}_\ell |-|\mathcal {K}_0|\le C_\mathrm{mesh}\sum _{j=0}^{\ell -1} |\mathcal M_j|\quad \text {for }\ell \in \mathbb {N}, \end{aligned}$$
  where $C_\mathrm{mesh}>0$ depends only on $[\mathcal {T}_0]$.

These assumptions are especially satisfied for pure *h*-refinement based on local bisection [1] as well as for the concrete strategy used in [24, 25]. The latter strategy looks as follows: Let $[\mathcal {T}_\star ]\in [\mathbb {T}]$. Let $\mathcal M_\star \subseteq \mathcal {N}_\star$ be a set of marked nodes. To get the refined mesh $[\mathcal {T}_{+}]:=\mathtt{ref}([\mathcal {T}_\star ],\mathcal M_\star )$, we proceed as follows:

(i) If both nodes of an element $T\in \mathcal {T}_\star$ belong to $\mathcal M_\star$, the element *T* will be marked.
(ii) For all other nodes in $\mathcal M_\star$, the multiplicity will be increased if it is less or equal to $p+1$, otherwise the elements which contain one of these nodes $z\in \mathcal M_\star$, will be marked.
(iii) Recursively, mark further elements $T'\in \mathcal {T}_\star$ for refinement if there exists a marked element $T\in \mathcal {T}_\star$ with $T\cap T'\ne \emptyset$ and $\check{h}_{\star ,T'}>\check{\kappa }_0 \check{h}_{\star ,T}$.
(iv) Refine all marked elements $T\in \mathcal {T}_\star$ by bisection and hence obtain $[\mathcal {T}_+]$.

According to [1], the proposed recursion in step (iii) terminates and the generated partition $\mathcal {T}_+$ guarantees (M1) with $\check{\kappa }_{\max }=2\check{\kappa }_0$. The following proposition shows that also the assumptions (M2)–(M3) are satisfied.

#### Proposition 2.2

The proposed refinement strategy $\mathtt{ref}(\cdot )$ used in [24, 25] satisfies Assumption 2.1, where $\check{\kappa }_{\max }=2\check{\kappa }_0$ and $C_\mathrm{mesh}$ depends only on the initial partition of the parameter domain,  i.e.,  $\mathcal {T}_0$ transformed onto [*a*, *b*].

#### Proof

For any partition $\mathcal {T}_\star$ of ${\varGamma }$ and any subset of marked elements $\mathcal {S}_\star \subseteq \mathcal {T}_\star$, let $\widetilde{\mathtt{ref}}(\mathcal {T}_\star ,\mathcal {S}_\star )$ be the partition obtained from the recursive bisection in step (iii)–(iv) above. This local *h*-refinement procedure has been analyzed in [1]. According to [1, Theorem 2.3], the recursion is well-defined and guarantees $\check{\kappa }_\star \le 2\check{\kappa }_0$ for all $\mathcal {T}_\star \in \widetilde{\mathtt{ref}}(\mathcal {T}_0)$.

To see (M2), [1, Theorem 2.3] guarantees the existence of some coarsest common refinement $\mathcal {T}_\star \widetilde{\oplus }\mathcal {T}_+\in \widetilde{\mathtt{ref}}(\mathcal {T}_\star )\cap \widetilde{\mathtt{ref}}(\mathcal {T}_+)$ such that

$$\begin{aligned} |\mathcal {T}_\star \widetilde{\oplus }\mathcal {T}_{+}|\le |\mathcal {T}_\star |+|\mathcal {T}_{+}|-|\mathcal {T}_0|. \end{aligned}$$

The corresponding nodes just satisfy $\mathcal {N}_\star \oplus \mathcal {N}_{+}=\mathcal {N}_\star \cup \mathcal {N}_{+}$. There exists a finite sequence of meshes $\mathcal {T}_\star ={\widetilde{\mathcal {T}}}_1,{\widetilde{\mathcal {T}}}_2={\widetilde{\mathtt{ref}}} ({\widetilde{\mathcal {T}}}_1,\mathcal {S}_1),\dots ,{\widetilde{\mathcal {T}}}_\ell ={\widetilde{\mathtt{ref}}} ({\widetilde{\mathcal {T}}}_{\ell -1},\mathcal {S}_{\ell -1})=\mathcal {T}_\star \widetilde{\oplus }\mathcal {T}_{+}$ with suitable $\mathcal {S}_j\subseteq \mathcal {T}_j$ for $j=1,\dots ,\ell -1$. If we define $\mathcal M_j\subseteq \mathcal {N}_j$ as the set of all nodes in $\mathcal {S}_j$, we see that the sequence $[\mathcal {T}_\star ]=[\mathcal {T}_1],[\mathcal {T}_2]=\mathtt{ref}([\mathcal {T}_1],\mathcal M_1),\dots [\mathcal {T}_\ell ]= \mathtt{ref}([\mathcal {T}_{\ell -1},\mathcal M_{\ell -1})$ satisfies $\mathcal {T}_j={\widetilde{\mathcal {T}}}_j$ for $j=1,\dots \ell$. By repetitively marking one single node, we obtain from $[\mathcal {T}_\ell ]$ a mesh $[\mathcal {T}_\star \oplus \mathcal {T}_+]$ with nodes $\mathcal {N}_\star \oplus \mathcal {N}_+=\mathcal {N}_\star \cup \mathcal {N}_+$ and $\# z=\max (\#_\star z,\#_+z)$, where $\#_\star$ resp. $\#_+$ denote the multiplicity in $\mathcal {K}_\star$ resp. $\mathcal {K}_+$ and, e.g., $\#_+ z:=0$ if $z\in \mathcal {N}_\star {\setminus }\mathcal {N}_+$. There obviously holds

$$\begin{aligned} |\mathcal {K}_\star \oplus \mathcal {K}_+|=\sum _{z\in \mathcal {N}_\star \cup \mathcal {N}_+} \# z\le |\mathcal {K}_\star |+|\mathcal {K}_+|-|\mathcal {K}_0|. \end{aligned}$$

Moreover, $[\mathcal {T}_\star \oplus \mathcal {T}_+]$ is clearly a refinement of $[\mathcal {T}_+]$ as well.

Finally we consider (M3). As before we have $\mathcal {T}_1={\widetilde{\mathtt{ref}}}(\mathcal {T}_0,\mathcal {S}_0),\dots ,$ $\mathcal {T}_\ell ={\widetilde{\mathtt{ref}}}(\mathcal {T}_{\ell -1},\mathcal {S}_{\ell -1})$ for suitable $\mathcal {S}_{j}\subseteq \mathcal {T}_j$, $j=0,\dots ,\ell -1$. Note that there holds $|\mathcal {S}_j|\le 2|\mathcal M_j|$. We denote $|\#_j|:=|\mathcal {K}_{j+1}|-|\mathcal {K}_{j}|-(|\mathcal {N}_{j+1}|-|\mathcal {N}_j|)$ as the number of multiplicity increases during the *j*-th refinement. There holds

$$\begin{aligned} |\mathcal {K}_{j+1}|-|\mathcal {K}_j|= |\mathcal {T}_{j+1}|-|\mathcal {T}_j|+|\#_j| \end{aligned}$$

and hence

$$\begin{aligned} |\mathcal {K}_\ell |-|\mathcal {K}_0|=|\mathcal {T}_\ell |-|\mathcal {T}_0|+\sum _{j=0}^{\ell -1} |\#_j|. \end{aligned}$$

The term $|\mathcal {T}_\ell |-|\mathcal {T}_0|$ can be estimated by $C\sum _{j=0}^{\ell -1} |\mathcal {S}_j|$ with some constant $C>0$ which depends only on the initial partition of the parameter domain, see [1, Theorem 2.3], and hence by $2C\sum _{j=0}^{\ell -1}|\mathcal M_j|$. The estimate $|\#_j|\le |\mathcal M_j|$ concludes the proof with $C_\mathrm{mesh}=2C+1$. $\square$

### B-splines and NURBS

**Fig. 2:**
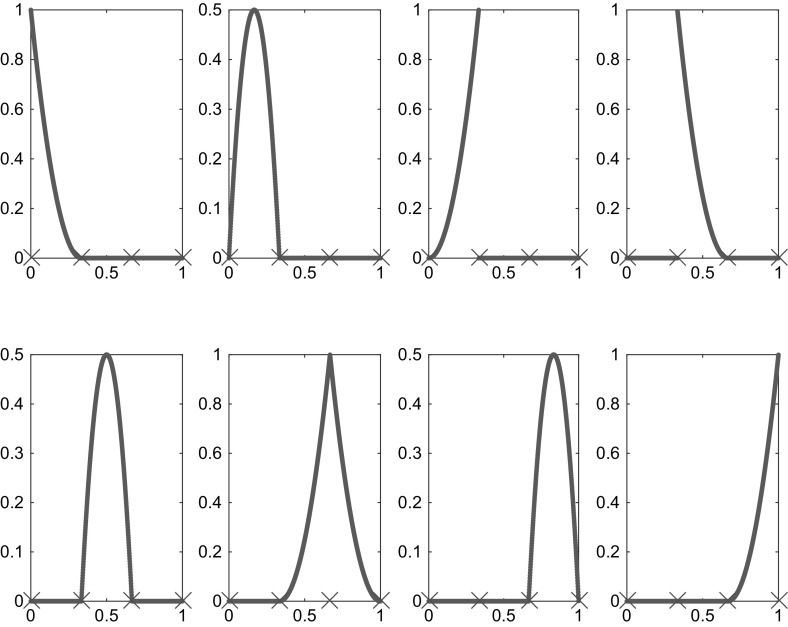
B-splines on the interval [0, 1] corresponding to knot sequence $(\dots ,0,0,0,1/3,1/3,1/3,2/3,2/3,1,1,1,\dots )$

Throughout this subsection, we consider *knots* $\check{\mathcal {K}}:=(t_i)_{i\in \mathbb {Z}}$ on $\mathbb {R}$ with multiplicity $\#t_i$ which satisfy $t_{i-1}\le t_{i}$ for $i\in \mathbb {Z}$ and $\lim _{i\rightarrow \pm \infty }t_i=\pm \infty$. Let $\check{\mathcal {N}}:=\big \{t_i\,:\,i\in \mathbb {Z}\big \}=\big \{\check{{z}}_{j}\,:\,j\in \mathbb {Z}\big \}$ denote the corresponding set of nodes with $\check{{z}}_{j-1}<\check{{z}}_{j}$ for $j\in \mathbb {Z}$. For $i\in \mathbb {Z}$, the *i*-th *B-spline* of degree *p* is defined inductively by (Fig. 2)

2.16 $$\begin{aligned}&B_{i,0}:=\chi _{[t_{i-1},t_{i})},\nonumber \\&B_{i,p}:=\beta _{i-1,p} B_{i,p-1}+(1-\beta _{i,p}) B_{i+1,p-1} \quad \text {for } p\in \mathbb {N}, \end{aligned}$$

where, for $$t\in \mathbb {R}$$,

$$\begin{aligned} \beta _{i,p}(t):= {\left\{ \begin{array}{ll} \frac{t-t_i}{t_{i+p}-t_i} &{}\text {if } t_i\ne t_{i+p},\\ 0 &{}\text {if } t_i= t_{i+p}. \end{array}\right. } \end{aligned}$$

We also use the notations $B_{i,p}^{\check{\mathcal {K}}}:=B_{i,p}$ and $\beta _{i,p}^{\check{\mathcal {K}}}:=\beta _{i,p}$ to stress the dependence on the knots $\check{\mathcal {K}}$. The following lemma collects some basic properties of B-splines.

#### Lemma 2.3

Let $$I=[a,b)$$ be a finite interval and $$p\in \mathbb {N}_0$$. Then,  the following assertions (i)–(vi) hold : 

(i) The set $\big \{B_{i,p}|_I\,:\,i\in \mathbb {Z}, B_{i,p}|_I\ne 0\big \}$ is a basis for the space of all right-continuous $\check{\mathcal {N}}$-piecewise polynomials of degree lower or equal *p* on *I* which are,  at each knot $t_i,$ $p-\#t_i$ times continuously differentiable if $p-\#t_i\ge 0$.
(ii) For $$i\in \mathbb {Z},$$ $$B_{i,p}$$ vanishes outside the interval $$[t_{i-1},t_{i+p})$$. It is positive on the open interval $$(t_{i-1},t_{i+p})$$.
(iii) For $i\in \mathbb {Z},$ $B_{i,p}$ is completely determined by the $p+2$ knots $t_{i-1},\dots ,t_{i+p}$.
(iv) The B-splines of degree *p* form a (locally finite) partition of unity,  i.e., 
  2.17 $$\begin{aligned} \sum _{i \in \mathbb {Z}} B_{i,p}=1\quad \text {on }\mathbb {R}. \end{aligned}$$

#### Proof

The proof of (i) is found in [14, Theorem 6], and (ii)–(iii) are proved in [14, Section 2]. (iv) is proved in [14, page 9–10]. $\square$

In addition to the knots $\check{\mathcal {K}}=(t_i)_{i\in \mathbb {Z}}$, we consider positive weights $\mathcal {W}:=(w_i)_{i\in \mathbb {Z}}$ with $w_i>0$. For $i\in \mathbb {Z}$ and $p\in \mathbb {N}_0$, we define the *i*-th NURBS by

2.18 $$\begin{aligned} R_{i,p}:=\frac{w_iB_{i,p}}{\sum _{\ell \in \mathbb {Z}} w_{\ell }B_{\ell ,p}}. \end{aligned}$$

We also use the notation $R_{i,p}^{\check{\mathcal {K}},\mathcal {W}}:=R_{i,p}$. Note that the denominator is locally finite and positive.

For any $p\in \mathbb {N}_0$, we define the B-spline space

2.19 $$\begin{aligned} \mathscr {S}^p(\check{\mathcal {K}}):=\left\{ \sum _{i\in \mathbb {Z}}a_i B_{i,p}:a_i\in \mathbb {R}\right\} \end{aligned}$$

as well as the NURBS space

2.20 $$\begin{aligned} \mathscr {N}^p(\check{\mathcal {K}},\mathcal {W}):=\left\{ \sum _{i\in \mathbb {Z}}a_i R_{i,p}:a_i\in \mathbb {R}\right\} =\frac{\mathscr {S}^p(\check{\mathcal {K}})}{\sum _{i\in \mathbb {Z}} w_\ell B_{\ell ,p}^{\check{\mathcal {K}}}}. \end{aligned}$$

### Ansatz spaces

Let $[\mathcal {T}_0]$ be a given initial mesh with corresponding knots $\mathcal {K}_0$ such that $h_0 \le |{\varGamma }|/4$ for closed ${\varGamma }=\partial {\varOmega }$. We set $[\mathbb {T}]:=\mathtt{ref}([\mathcal {T}_0])$. Suppose that $\mathcal {W}_0=(w_{i})_{i=1-p}^{N-p}$ are given initial weights with $N=|\mathcal {K}_0|$ for closed ${\varGamma }=\partial {\varOmega }$ resp. $N=|\mathcal {K}_0|-(p+1)$ for open ${\varGamma }\subsetneqq \partial {\varOmega }$.

If ${\varGamma }=\partial {\varOmega }$ is closed, we extend the transformed knot sequence $\check{\mathcal {K}}_0=(t_i)_{i=1}^N$ arbitrarily to $(t_i)_{i\in \mathbb {Z}}$ with $t_{-p}=\dots =t_0=a$, $t_i\le t_{i+1}$, $\lim _{i\rightarrow \pm \infty }t_i=\pm \infty$ and $\mathcal {W}_0=(w_i)_{i\in \mathbb {Z}}$ with $w_i>0$. For the extended sequences, we also write $\check{\mathcal {K}}_0$ and $\mathcal {W}_0$ and set

2.21 $$\begin{aligned} \mathcal {X}_{0}:=\mathscr {N}^p(\check{\mathcal {K}}_{0},\mathcal {W}_{0})|_{[a,b)}\circ \gamma |_{[a,b)}^{-1}. \end{aligned}$$

If ${\varGamma }_{}\subsetneqq \partial {\varOmega }$ is open, we extend the sequences $\check{\mathcal {K}}_0=(t_i)_{i=-p}^N$ and $\mathcal {W}_0$ arbitrarily to $(t_i)_{i\in \mathbb {Z}}$ with $t_i\le t_{i+1}$, $\lim _{i\rightarrow \pm \infty }t_i=\pm \infty$ and $\mathcal {W}_0=(w_i)_{i\in \mathbb {Z}}$ with $w_i>0$. This allows to define

2.22 $$\begin{aligned} \mathcal {X}_0:=\mathscr {N}^p(\check{\mathcal {K}}_0,\mathcal {W}_0)|_{[a,b]}\circ \gamma ^{-1}. \end{aligned}$$

Due to Lemma 2.3, this definition does not depend on how the sequences are extended.

Let $[\mathcal {T}_\star ]\in [\mathbb {T}]$ be a mesh with knots $\mathcal {K}_\star$. Via *knot insertion* from $\mathcal {K}_0$ to $\mathcal {K}_\star$, one obtains unique corresponding weights $\mathcal {W}_\star$. These are chosen such that the denominators of the NURBS functions do not change. In particular, this implies nestedness

2.23 $$\begin{aligned} \mathcal {X}_\star \subseteq \mathcal {X}_{+} \quad \text {for all }[\mathcal {T}_\star ]\in [\mathbb {T}], [\mathcal {T}_{+}]\in \mathtt{ref}(\mathcal {T}_\star ), \end{aligned}$$

where the spaces $\mathcal {X}_\star$ resp. $\mathcal {X}_+$ are defined analogously to (2.21)–(2.22). Moreover, the weights are just convex combinations of $\mathcal {W}_0$, wherefore

2.24 $$\begin{aligned} w_{\min }:=\min (\mathcal {W}_0)\le \min (\mathcal {W}_\star )\le \max (\mathcal {W}_\star )\le \max (\mathcal {W}_0)=:w_{\max }. \end{aligned}$$

For further details, we refer to, e.g., [25, Section 4.2].

## Adaptive algorithm and main results

For each mesh $[\mathcal {T}_\star ]\in [\mathbb {T}]$, define the node-based error estimator

3.1a $$\begin{aligned} \mu _{\star }^2=\sum _{z\in \mathcal {N}_\star } \mu _{\star }(z)^2, \end{aligned}$$

where the refinement indicators read

3.1b $$\begin{aligned} \mu _{\star }(z)^2:= |\gamma ^{-1}({\omega }_{\star }(z))| \Vert \partial _{\varGamma }(f-V{\varPhi }_\star )\Vert _{L^2(\omega _{\star }(z))}^2 \quad \text {for all }z\in \mathcal {N}_\star . \end{aligned}$$

Here, we must additionally suppose $f\in H^{1}({\varGamma })$ to ensure that $\mu _\star$ is well-defined. It has been proved in [24] that $\mu _\star$ is reliable, i.e.,

3.2 $$\begin{aligned} \Vert \phi -{\varPhi }_\star \Vert _{\widetilde{H}^{-1/2}({\varGamma })} \le C_\mathrm{rel}\,\mu _\star , \end{aligned}$$

where $C_\mathrm{rel}>0$ depends only on *p*, $w_\mathrm{min}$, $w_\mathrm{max}$, $\gamma$, and $\check{\kappa }_\mathrm{max}$. We note that the weighted-residual error estimator in the form $\mu _\star \simeq \Vert h_\star ^{1/2}\partial _{\varGamma }(f-V{\varPhi }_\star )\Vert _{L^2({\varGamma })}$ goes back to the works [6, 13], where reliability (3.2) is proved for standard 2D BEM with piecewise constants on polyhedral geometries, while the corresponding result for 3D BEM is found in [12]. We consider the following adaptive algorithm which employs the Dörfler marking strategy (3.3) from [17] to single out nodes for refinement.

### Algorithm 3.1

**Input:** Adaptivity parameter $0<\theta <1$, $C_\mathrm{mark}\ge 1$, polynomial order $p\in \mathbb {N}_0$, initial mesh $[\mathcal {T}_0]$, initial weights $\mathcal {W}_0$.

**Adaptive loop:** For each $\ell =0,1,2,\dots$ iterate the following steps (i)–(iv):

(i) Compute discrete approximation ${\varPhi }_\ell \in \mathcal {X}_\ell$ from Galerkin BEM.
(ii) Compute refinement indicators $\mu _\ell ({z})$ for all nodes ${z}\in \mathcal {N}_\ell$.
(iii) Determine an up to the multiplicative constant $C_\mathrm{mark}$ minimal set of nodes $\mathcal M_\ell \subseteq \mathcal {N}_\ell$ such that
  3.3 $$\begin{aligned} \theta \,\mu _\ell ^2 \le \sum _{{z}\in \mathcal M_\ell }\mu _\ell ({z})^2. \end{aligned}$$
(iv) Generate refined mesh $[\mathcal {T}_{\ell +1}]:=\mathtt{ref}([\mathcal {T}_\ell ],\mathcal M_\ell )$.

**Output:** Approximate solutions ${\varPhi }_\ell$ and error estimators $\mu _\ell$ for all $\ell \in \mathbb {N}_0$.

Our main result is that the proposed algorithm is linearly convergent, even with the optimal algebraic rate. For a precise statement of this assertion, let $[\mathbb {T}_N]:=\big \{[\mathcal {T}_\star ]\in [\mathbb {T}]\,:\,|\mathcal {K}_\star |-|\mathcal {K}_0|\le N\big \}$ be the finite set of all refinements having at most *N* knots more than $[\mathcal {T}_0]$. Following [8], we introduce an estimator-based approximation class $\mathbb {A}_s$ for $s>0$: We write $\phi \in \mathbb {A}_s$ if

3.4 $$\begin{aligned} \Vert \phi \Vert _{\mathbb {A}_s}:=\sup _{N\in \mathbb {N}_0} \left( (N+1)^s \min _{[\mathcal {T}_\star ]\in [\mathbb {T}_N]} \mu _\star \right) <\infty . \end{aligned}$$

In explicit terms, this just means that an algebraic convergence rate of $\mathcal {O}(N^{-s})$ for the estimator is possible, if the optimal meshes are chosen. The following theorem is the main result of our work:

### Theorem 3.2

Let $f\in H^1({\varGamma }),$ so that the weighted-residual error estimator $\mu _\ell$ from (3.1) is well-defined and that Algorithm 3.1 is driven by $\mu _\ell .$ We suppose that the Assumption 2.1 on the mesh-refinement holds true. Then,  for each $0<\theta \le 1,$ there exist constants $0<q_\mathrm{lin}<1$ and $C_\mathrm{lin}>0$ such that Algorithm 3.1 is linearly convergent in the sense of

3.5 $$\begin{aligned} \mu _{\ell +n}\le C_\mathrm{lin}\,q_\mathrm{lin}^n\,\mu _\ell \quad \text {for all }\ell ,n\in \mathbb {N}_0. \end{aligned}$$

In particular,  this implies convergence

3.6 $$\begin{aligned} C_\mathrm{rel}^{-1}\,\Vert \phi -{\varPhi }_\ell \Vert _{\widetilde{H}^{-1/2}({\varGamma })} \le \mu _\ell \le C_\mathrm{lin}q_\mathrm{lin}^\ell \,\mu _0 \xrightarrow {\ell \rightarrow \infty }0. \end{aligned}$$

Moreover,  there is a constant $0<\theta _\mathrm{opt}<1$ such that for all $0<\theta <\theta _\mathrm{opt},$ there exists a constant $C_\mathrm{opt}>0$ such that,  for all $s>0,$ it holds

3.7 $$\begin{aligned} \phi \in \mathbb {A}_s\quad \Longrightarrow \quad \mu _\ell \le \frac{C_\mathrm{opt}^{1+s}}{(1-q_\mathrm{lin}^{1/s})^s} \Vert \phi \Vert _{\mathbb {A}_s}(|\mathcal {K}_\ell |-|\mathcal {K}_0|)^{-s}\quad \text {for all }\ell \in \mathbb {N}_0. \end{aligned}$$

The constants $q_\mathrm{lin}, C_\mathrm{lin}$ depend only on $p, w_{\min },w_{\max }, \gamma , \theta ,$ and $\check{\kappa }_{\max }$ from (M1). The constant $\theta _\mathrm{opt}$ depends only on $p, w_{\min },w_{\max }, \gamma ,$ and (M1)–(M3), and $C_\mathrm{opt}$ depends additionally on $\theta$.

### Remark 3.3

The proof of Theorem 3.2 reveals that linear convergence (3.5) only requires (M1), while optimal rates (3.7) rely on (3.5) and (M2)–(M3). Provided that there exists a constant $C_\mathrm{son} > 0$ such that $|\mathcal {K}_\star |\le C_\mathrm{son}|\mathtt{ref}([\mathcal {T}_\star ],\mathcal M_\star )|$ for all $[\mathcal {T}_\star ]\in [\mathbb {T}]$ and $\mathcal M_\star \subseteq \mathcal {N}_\star$, also the converse implication in (3.7) holds true. The proof follows along the lines of [8, Proposition 4.15] and thus is left to the reader.

The proof of Theorem 3.2 is given in Sects. 4 and 5. The ideas essentially follow those of [8], where an axiomatic approach of adaptivity for abstract problems is found. We note, however, that [8] only considers *h*-refinement, while the present formulation of Algorithm 3.1 steers both, the *h*-refinement and the knot multiplicity increase.

If Algorithm 3.1 is steered by the Faermann estimator

3.8a $$\begin{aligned} \eta _{\star }^2=\sum _{z\in \mathcal {N}_\star } \eta _{\star }(z)^2 \end{aligned}$$

with the refinement indicators

3.8b $$\begin{aligned} \eta _{\star }(z)^2:= |f-V{\varPhi }_\star |_{H^{1/2}(\omega _{\star }(z))}^2 \quad \text {for all }z\in \mathcal {N}_\star , \end{aligned}$$

instead of $\mu _\star$, we can prove at least plain convergence of the estimator to zero. In contrast to the weighted-residual estimator which requires additional regularity $f\in H^1({\varGamma })$, the Faermann estimator $\eta _\star$ allows a right-hand side $f\in H^{1/2}({\varGamma })$. Moreover, $\eta _\star$ estimator is efficient and reliable

3.9 $$\begin{aligned} C_\mathrm{eff}^{-1} \eta _\star \le \Vert \phi -{\varPhi }_\star \Vert _{\widetilde{H}^{-1/2}({\varGamma })} \le C_\mathrm{rel}\,\eta _\star , \end{aligned}$$

where $C_\mathrm{eff}>0$ depends only on ${\varGamma }$, while $C_\mathrm{rel}>0$ depends additionally on $p, \check{\kappa }_{\max }, w_{\min }, w_{\max }$ and $\gamma$; see [25, Theorem 3.1 and 4.4]. This equivalence of error and estimator puts some interest on the following convergence theorem which is, however, weaker than the statement of Theorem 3.2.

### Theorem 3.4

Let $f\in H^{1/2}({\varGamma })$. We suppose that (M1) from Assumption 2.1 for the mesh-refinement holds. Then,  for each $0<\theta \le 1,$ Algorithm 3.1 steered by the Faermann estimator (3.8) is convergent in the sense of

3.10 $$\begin{aligned} \eta _\ell \xrightarrow {\ell \rightarrow \infty }0. \end{aligned}$$

According to (3.9),  this is equivalent to

3.11 $$\begin{aligned} \Vert \phi -{\varPhi }_\ell \Vert _{\widetilde{H}^{-1/2}({\varGamma })} \xrightarrow {\ell \rightarrow \infty }0. \end{aligned}$$

### Remark 3.5

The statements of Theorems 3.2 and 3.4 remain valid, if only adaptive *h*-refinement is used, i.e., if Algorithm 3.1 does not steer the knot multiplicity.

## Proof of Theorem 3.2, linear convergence (3.5)

As an auxiliary result, we need an inverse-type estimate for NURBS with respect to the fractional $\widetilde{H}^{-1/2}({\varGamma })$-norm. In the following, a result is stated and proved for the $\widetilde{H}^{-\sigma }({\varGamma })$-norm, where $0<\sigma <1$. For piecewise polynomials, an analogous result is already found in [30, Theorem 3.6] resp. [29, Theorem 3.9]. Our proof is inspired by [15, Section 4.3], where a similar result is found for piecewise constant functions as well as for piecewise affine and globally continuous functions in 1D. For integer-order Sobolev norms, inverse estimates for NURBS are found in [3, Section 4], and (4.2) is proved in [2, Theorem 3.1] for piecewise polynomials.

### Proposition 4.1

Let $[\mathcal {T}_\star ]\in [\mathbb {T}]$ and $0<\sigma <1$. Then,  there is a constant $C_\mathrm{inv}>0$ such that

4.1 $$\begin{aligned} \Vert h_\star ^{\sigma }{\varPsi }_\star \Vert _{L^2({\varGamma })}\le C_\mathrm{inv}\Vert {\varPsi }_\star \Vert _{\widetilde{H}^{-\sigma }({\varGamma })}\quad \text {for all } {\varPsi }_\star \in \mathcal {X}_\star . \end{aligned}$$

For $\sigma =1/2,$ it holds

4.2 $$\begin{aligned} \Vert h_\star ^{1/2}\partial _{\varGamma } (V{\varPsi }_\star )\Vert _{L^2({\varGamma })}+\Vert h_\star ^{1/2}{\varPsi }_\star \Vert _{L^2({\varGamma })}\le C_\mathrm{inv}\Vert {\varPsi }_\star \Vert _{\widetilde{H}^{-1/2}({\varGamma })}\quad \text {for all } {\varPsi }_\star \in \mathcal {X}_\star .\nonumber \\ \end{aligned}$$

The constant $C_\mathrm{inv}$ only depends on $\check{\kappa }_{\max }, p, w_{\min }, w_{\max },$ $\gamma ,$ and $\sigma$.

### Proof

The proof is done in four steps. First, we show that $\Vert h_\star ^{\sigma }\psi \Vert _{L^2({\varGamma })}\lesssim \Vert \psi \Vert _{\widetilde{H}^{-\sigma }({\varGamma })}$ holds for all $\psi \in L^2({\varGamma })$ which satisfy a certain assumption. In the second step, we prove an auxiliary result for polynomials which is needed in the third one, where we show that all $\psi \in \mathcal {X}_\star$ satisfy the mentioned assumption. In the last step, we apply a recent result of [2], which then concludes the proof.

*Step 1* Let $\mathcal {X}\subset L^2({\varGamma })$ satisfy the following assumption: There exists a constant $q \in (0,1)$ such that for all $T\in \mathcal {T}_\star$ and all $\psi \in \mathcal {X}$ there exists some connected subset ${\varDelta }(T,\psi ) \subseteq T$ of length $|{\varDelta }(T,\psi )|\ge q|T|$ such that $\psi$ does not change its sign on ${\varDelta }(T,\psi )$ and

4.3 $$\begin{aligned} \min _{x\in {\varDelta }(T,\psi )}|\psi (x)|\ge q \,\max _{x\in T} |\psi (x)|. \end{aligned}$$

Then, there exists a constant $C>0$ which depends only on *q* and $\check{\kappa }_\star$, such that

$$\begin{aligned} \Vert h_\star ^{\sigma } \psi \Vert _{L^2({\varGamma })} \le C \Vert \psi \Vert _{\widetilde{H}^{-\sigma }({\varGamma })} \quad \text {for all }\psi \in \mathcal {X}. \end{aligned}$$

For a compact nonempty interval $[c,d]= I \subseteq [a,b]$, we define the bubble function

$$\begin{aligned} P_I(t):= {\left\{ \begin{array}{ll} \left( \frac{t-c}{d-c}\cdot \frac{d-t}{d-c}\right) ^2\quad &{}\text {if } t\in I, \\ 0 \quad &{}\text {if }t\in [a,b]{\setminus } I. \end{array}\right. } \end{aligned}$$

It obviously satisfies $0\le P_I\le 1$ and $\mathrm{supp}P_I=I$. A standard scaling argument proves

4.4 $$\begin{aligned} C_1|I|\le \Vert P_I\Vert _{L^2(I)}^2 \le \Vert P_I\Vert _{L^1(I)} \le C_2 |I| \end{aligned}$$

and

4.5 $$\begin{aligned} |I|^2\Vert P_I'\Vert _{L^2(I)}^2\le C_3\Vert P_I\Vert _{L^2(I)}^2 \end{aligned}$$

with generic constants $C_1,C_2,C_3>0$ which do not depend on *I*. For each $T\in \mathcal {T}_\star$, let $I(T,\psi )$ be some interval with $\gamma (I(T,\psi ))={\varDelta }(T,\psi )$. With the arclength parametrization $\gamma _L$, we define, for all $T\in \mathcal {T}_\star$, the functions $P_{{\varDelta }(T,\psi )}:=P_{I(T,\psi )}\circ \gamma _L$ and the coefficients

4.6 $$\begin{aligned} c_T:={\mathrm {sgn}}(\psi |_{{\varDelta }(T,\psi )})h_{\star ,T}^{2\sigma } \min _{x\in {\varDelta }(T,\psi )} |\psi (x)|. \end{aligned}$$

Note that (4.4) and (4.5) hold for $P_{{\varDelta }(T,\psi )}$ with *I* simply replaced by ${\varDelta }(T,\psi )$ and with $(\cdot )'$ replaced by the arclength derivative $\partial _{\varGamma }$. By definition of the dual norm, it holds

4.7 $$\begin{aligned} \Vert \psi \Vert _{\widetilde{H}^{-\sigma }({\varGamma })}\ge \frac{|\langle \psi \,;\,\chi \rangle |}{\Vert \chi \Vert _{H^{\sigma }({\varGamma })}} \quad \text {with, e.g., } \chi := \sum _{T\in \mathcal {T}_\star } c_T P_{{\varDelta }(T,\psi )}\in H^1({\varGamma })\subset H^{\sigma }({\varGamma }).\nonumber \\ \end{aligned}$$

First, we estimate the numerator in (4.7):

$$\begin{aligned} |\langle \psi \,;\,\chi \rangle |&= \left| \sum _{T\in \mathcal {T}_\star } \int _{T} \psi (x) c_T P_{{\varDelta }(T,\psi )}(x) \,dx\right| \\&\mathop {=}\limits ^{(4.6)} \sum _{T\in \mathcal {T}_\star } h_{\star ,T}^{2\sigma } \min _{x\in {\varDelta }(T,\psi )}|\psi (x)|^2\,\Vert P_{{\varDelta }(T,\psi )}\Vert _{L^1({\varDelta }(T,\psi ))}\\&\mathop {\ge }\limits ^{(4.3)} {q^2} \sum _{T\in \mathcal {T}_\star } h_{\star ,T}^{2\sigma }\max _{x\in T} |\psi (x)|^2 {\Vert P_{{\varDelta }(T,\psi )}\Vert _{L^1({\varDelta }(T,\psi ))}}\\&\mathop {\ge }\limits ^{(4.4)} {C_1q^3 \sum _{T\in \mathcal {T}_\star } h_{\star ,T}^{2\sigma }\,\Vert \psi \Vert _{L^2(T)}^2 }\\&= C_1 q^3 \Vert h_\star ^{\sigma }\psi \Vert _{L^2({\varGamma })}^2. \end{aligned}$$

It remains to estimate the denominator in (4.7): We first note that it holds $|u|_{H^{\sigma }(I)}^2\lesssim |I|^{1-\sigma }\Vert u'\Vert _{L^2(I)}$ for any interval $I\subset \mathbb {R}$ of finite length and $u\in H^1(I)$. This is already stated in [7, Lemma 7.4]. However, a detailed proof is given only for $1/2<\sigma <1$. For $0<\sigma \le 1/2$ this inequality can be shown exactly as in the proof of [24, Lemma 4.5], where only $\sigma =1/2$ is considered. This, together with (2.8), implies for any connected $\omega \subseteq {\varGamma }$ with $|\omega |\le \frac{3}{4}|{\varGamma }|$ that

4.8 $$\begin{aligned} |u|_{H^{\sigma }(\omega )}\lesssim |\omega |^{1-\sigma } \Vert \partial _{\varGamma } u\Vert _{L^2(\omega )}\quad \text {for all } u\in H^1({\varGamma }). \end{aligned}$$

The hidden constant in (4.8) depends only on $\sigma$ and ${\varGamma }$. Equation (4.8) is applicable for any node patch $\omega _\star (z)$ since we assumed in Sect. 2.8 that $h_0\le |{\varGamma }|/4$ if ${\varGamma }=\partial {\varOmega }$ With [18, Lemma 2.3], we hence see

$$\begin{aligned} |\chi |_{H^{\sigma }({\varGamma })}^2&\mathop {\lesssim }\limits ^{[18]}\Vert h_{\star }^{-\sigma }\chi \Vert _{L^2({\varGamma })}^2+\sum _{z\in \mathcal {N}_\star } |\chi |_{H^{\sigma }(\omega _z)}^2 \\&{\mathop {\lesssim }\limits ^{(4.8)}} \Vert h_{\star }^{-\sigma }\chi \Vert _{L^2({\varGamma })}^2+\sum _{z\in \mathcal {N}_\star } \Vert h_\star ^{1-\sigma } \partial _{\varGamma }\chi \Vert _{L^2(\omega _z)}^2 \\&\simeq \Vert h_{\star }^{-\sigma }\chi \Vert _{L^2({\varGamma })}^2+\sum _{T\in \mathcal {T}_\star } \Vert h_\star ^{1-\sigma } \partial _{\varGamma }\chi \Vert _{L^2({\varDelta }(T,\psi ))}^2\\&=\Vert h_\star ^{-\sigma }\chi \Vert _{L^2({\varGamma })}^2 + \sum _{T\in \mathcal {T}_\star } h_{\star ,T}^{2-2\sigma }c_T^2 \Vert \partial _{\varGamma } P_{{\varDelta }(T,\psi )}\Vert _{L^2({\varDelta }(T,\psi ))}^2\\&\mathop {\le }\limits ^{(4.5)}{\Vert h_\star ^{-\sigma }\chi \Vert _{L^2({\varGamma })}^2}+C_3\sum _{T\in \mathcal {T}_\star } h_{\star ,T}^{2-2\sigma }c_T^2|{\varDelta }(T,\psi )|^{-2} \Vert P_{{\varDelta }(T,\psi )}\Vert _{L^2({\varDelta }(T,\psi ))}^2\\&\simeq \Vert h_\star ^{-\sigma }\chi \Vert _{L^2({\varGamma })}^2. \end{aligned}$$

This yields

$$\begin{aligned} \Vert \chi \Vert _{H^{\sigma }({\varGamma })}^2 = {\Vert \chi \Vert _{L^2({\varGamma })}^2 + |\chi |_{H^{\sigma }({\varGamma })}^2} \lesssim \Vert h_\star ^{-\sigma } \chi \Vert _{L^2({\varGamma })}^2, \end{aligned}$$

where the hidden constant depends only on $\check{\kappa }_{\max }, \sigma$, and $\gamma$. With

$$\begin{aligned} \Vert h_\star ^{-\sigma } \chi \Vert _{L^2({\varGamma })}^2&=\sum _{T\in \mathcal {T}_\star } h_{\star ,T}^{-2\sigma } c_T ^2\Vert P_{{\varDelta }(T,\psi )}\Vert _{L^2({\varDelta }(T,\psi ))}^2 \mathop {\le }\limits ^{(4.4)} C_2\sum _{T\in \mathcal {T}_\star }h_{\star ,T}^{-2\sigma } c_T^2 |{\varDelta }(T,\psi )|\\&\mathop {=}\limits ^{(4.6)} C_2\sum _{T\in \mathcal {T}_\star } h_{\star ,T}^{2\sigma }\min _{x\in {\varDelta }(T,\psi )} |\psi (x)|^2|{\varDelta }(T,\psi )| \\&\le {C_2 \sum _{T\in \mathcal {T}_\star } h_{\star ,T}^{2\sigma } \Vert \psi \Vert _{L^2({\varDelta }(T,\psi ))}^2}\le C_2 \Vert h_\star ^{\sigma } \psi \Vert _{L^2({\varGamma })}^2, \end{aligned}$$

we finish the first step.

*Step 2* For some fixed polynomial degree $p\in \mathbb {N}_0$, there exists a constant $q_1\in (0,1)$ such that for all polynomials *F* of degree *p* on [0, 1] there exists some interval $I\subseteq [0,1]$ of length $|I|\ge q_1$ with

4.9 $$\begin{aligned} \min _{t\in I}|F(t)|\ge q_1 \,\max _{t\in [0,1]} |F(t)|. \end{aligned}$$

Instead of considering general polynomials $\mathcal {P}^p([0,1])$ of degree *p*, it is sufficient to consider the following subset

$$\begin{aligned} \mathcal {M}:=\big \{F\in \mathcal {P}^p([0,1])\,:\,\Vert F\Vert _{\infty }=1\big \}. \end{aligned}$$

Note that $\mathcal {M}$ is a compact subset of $L^{\infty }([0,1])$ and that differentiation $(\cdot )'$ is a continuous mapping on $\mathcal {M}$ due to finite dimension. This especially implies boundedness $\sup _{F\in \mathcal {M}} \Vert F'\Vert _{\infty }\le C_4<\infty$. We may assume $C_4>2$. For given $F\in \mathcal {M}$, we define the interval *I* as follows: Without loss of generality, we assume that the maximum of |*F*| is attained at some $t_1\in [0,1/2]$ and that $F(t_1)=1$. We set $t_3:=t_1+C_4^{-1}\in (t_1,1]$ and $t_2:=t_1+C_4^{-1}/2\in (t_1,3/4]$ and $I:=[t_1,t_2]$. Then, $|I|=1/(2C_4)$ and for all $t\in I$ it holds

$$\begin{aligned} 1/2\le C_4(t_3-t)=F(t_1)+C_4(t_1-t)\le F(t_1)+\Vert F'\Vert _{\infty }(t_1-t)\le F(t)=|F(t)|. \end{aligned}$$

Altogether, we thus have

$$\begin{aligned} q_1:= 1/(2C_4)\le 1/2 \le \min _{t\in I} |F(t)| \quad \text {and} \quad |I|=q_1 \end{aligned}$$

and conclude this step.

*Step 3* We show that $\mathcal {X}_\star$ satisfies the assumption of Step 1 and hence conclude $\Vert h_\star ^{\sigma }{\varPsi }_\star \Vert _{L^2({\varGamma })}\lesssim \Vert {\varPsi }_\star \Vert _{\widetilde{H}^{-\sigma }({\varGamma })}$ for all ${\varPsi }_\star \in \mathcal {X}_\star$: Let $\check{T}\subset [a,b]$ be the interval with $\gamma (\check{T})=T$ and $\check{\psi }:=\psi \circ \gamma |_{\check{T}}$. Since $|I|\simeq |\gamma (I)|$ for any interval $I\subseteq [a,b]$, where the hidden constants depend only on $\gamma$, we just have to find a uniform constant $q_2\in (0,1)$ and some interval $I\subseteq \check{T}$ of length $|I|\ge q_2 |\check{T}|$ with

4.10 $$\begin{aligned} \min _{t\in I}|\check{\psi }(t)|\ge q_2 \max _{x\in \check{T}} |\check{\psi }(t)|. \end{aligned}$$

The function $\check{\psi }$ has the form *F* / *w* with a polynomial *F* of degree *p* and the weight function *w*, which is also a polynomial of degree *p* and which satisfies $w_{\min }\le w\le w_{\max }$. Hence, (4.10) is especially satisfied if

4.11 $$\begin{aligned} \min _{t\in I}|F(t)|\ge q_1\,\frac{w_{\max }}{w_{\min }} \, \max _{x\in \check{T}} |F(t)|. \end{aligned}$$

After scaling to the interval [0, 1], we can apply Step 2 and conclude this step. Altogether, this proves (4.1).

*Step 4* According to [2], it holds $\Vert h_\star ^{1/2}\partial _{\varGamma }(V\psi )\Vert _{L^2({\varGamma })}\lesssim \Vert h_\star ^{1/2}\psi \Vert _{L^2({\varGamma })}+\Vert \psi \Vert _{\widetilde{H}^{-1/2}({\varGamma })}$ for all $\psi \in L^2({\varGamma })$, where the hidden constant depends only on ${\varGamma }$, $\gamma$, and $\check{\kappa }_{\max }$. Together with Step 3, this shows (4.2). $\square$

The proof of linear convergence (3.5) will be done with the help of some auxiliary (and purely theoretical) error estimator ${\widetilde{\rho }}_\star$. The latter relies on the following definition of an equivalent mesh-size function which respects the multiplicity of the knots.

### Proposition 4.2

Assumption 2.1 (M1) implies the existence of a modified mesh-size function $\widetilde{h}:[\mathbb {T}]\rightarrow L^\infty ({\varGamma })$ with the following properties :  There exists a constant $C_\mathrm{wt}>0$ and $0<q_\mathrm{ctr}<1$ which depend only on $\check{\kappa }_{\max }, p$ and $\gamma$ such that for all $[\mathcal {T}_\star ]\in [\mathbb {T}]$ and all refinements $[\mathcal {T}_{+}]\in \mathtt{ref}([\mathcal {T}_\star ]),$ the corresponding mesh-sizes $\widetilde{h}_\star :=\widetilde{h}([\mathcal {T}_\star ])$ and $\widetilde{h}_{+}:=\widetilde{h}([\mathcal {T}_{+}])$ satisfy equivalence

4.12 $$\begin{aligned} C_\mathrm{wt}^{-1}\check{h}_\star \le \widetilde{h}_\star \le C_\mathrm{wt}\check{h}_\star , \end{aligned}$$

reduction

4.13 $$\begin{aligned} \widetilde{h}_{+}\le \widetilde{h}_\star , \end{aligned}$$

as well as contraction on the patch of refined elements

4.14 $$\begin{aligned} \widetilde{h}_{+}|_{\omega _{+}([\mathcal {T}_+]{\setminus } [\mathcal {T}_\star ])}\le q_\mathrm{ctr}\widetilde{h}_\star |_{\omega _{+}([\mathcal {T}_+]{\setminus } [\mathcal {T}_\star ])}. \end{aligned}$$

Note that $\omega _{+}( [\mathcal {T}_{+}]{\setminus }[\mathcal {T}_{\star }])=\omega _{{\star }}( [\mathcal {T}_{\star }]{\setminus }[\mathcal {T}_{+}]),$ which follows from $\bigcup ([\mathcal {T}_+]{\setminus } [\mathcal {T}_\star ])=\bigcup ([\mathcal {T}_\star ]{\setminus }[\mathcal {T}_+])$ and the fact that the application of $\omega _{+}$ resp. $\omega _{\star }$ only adds elements of $\mathcal {T}_{\star }{\cap {\mathcal {T}}}_{+}$.

### Proof

For all $[\mathcal {T}_\star ]\in \mathbb {T}$, we define $\widetilde{h}_\star \in L^\infty ({\varGamma })$ by

$$\begin{aligned} \widetilde{h}_\star |_T=|\gamma ^{-1}(\omega _\star (T))| \cdot q_1^{\sum _{z\in \mathcal {N}_\star {\cap {\omega }}_\star (T)}\#z} \quad \text {for all }T\in \mathcal {T}_\star , \end{aligned}$$

where $0<q_1<1$ is fixed later. Clearly, $\widetilde{h}_\star \simeq \check{h}_\star$, where the hidden equivalence constants depend only on $\check{\kappa }_\star$, *p*, and $q_1$. Let $x\in {\varGamma }$. First, suppose $x\not \in \omega _{+}([\mathcal {T}_{+}]{\setminus }[\mathcal {T}_\star ])\cup \mathcal {N}_{+}$, i.e., neither the element $[T]\in [\mathcal {T}_{+}]$ containing *x* nor its neighbors result from *h*-refinement or from multiplicity increase. Then, $\widetilde{h}_{+}(x)=\widetilde{h}_\star (x)$. Second, suppose $x\in \omega _{+}([\mathcal {T}_{+}]{\setminus }[\mathcal {T}_\star ]){\setminus }\mathcal {N}_{+}$, i.e., the element $[T']\in [\mathcal {T}_{+}]$ containing *x* or one of its neighbors result from *h*-refinement and/or multiplicity increase. If only multiplicity increase took place, we get

$$\begin{aligned} q_1^{\sum _{z\in \mathcal {N}_+{\cap {\omega }}_+(T')} \# z}\le q_1\cdot q_1^{\sum _{z\in \mathcal {N}_\star {\cap {\omega }}_\star (T)} \# z}. \end{aligned}$$

In the other case, consider the father $[T]\in [\mathcal {T}_\star ]$ of $[T']$, i.e., $T'\subseteq T$. Note that

$$\begin{aligned} |\gamma ^{-1}(\omega _{+}(T'))|\le q_2\, |\gamma ^{-1}(\omega _\star (T))| \end{aligned}$$

with a constant $0<q_2<1$ which depends only on $\check{\kappa }_{\max }$. Choose $0<q_1<1$ sufficiently large such that

$$\begin{aligned} {q_2}/{q_1^{4p}}<1. \end{aligned}$$

This choice yields $\widetilde{h}_{+}(x)\le ({q_2}/q_1^{4p}) \cdot \widetilde{h}_\star (x)$, since $\mathcal {N}_\star {\cap {\omega }}_\star (T)$ contains at most 4 nodes. Therefore, we conclude the proof with $q_\mathrm{ctr}:=\max (q_1,{q_2}/q_1^{4p})$. $\square$

### Remark 4.3

Note that the construction of $\widetilde{h}_\star$ in Proposition 4.2 even ensures contraction $\widetilde{h}_{+}|_{\omega _{+}(T)}\le q_\mathrm{ctr}\widetilde{h}_\star |_{\omega _{+}(T)}$ if ${[T]\in [\mathcal {T}_{+}]{\setminus } [\mathcal {T}_\star ]}$ is obtained by *h*-refinement, while the multiplicity of all nodes $z\in \mathcal {N}_+{\cap {\omega }}_+(T)$ is arbitrarily chosen $\#z\in \{1,\dots ,p+1\}$. In explicit terms, this allows for instance to set the multiplicity of all nodes $z\in \mathcal {N}_+{\cap {\omega }}_+(T)$ to $\#z:=1$, if *T* is obtained by *h*-refinement.$\square$

For any $[\mathcal {T}_\star ]\in [\mathbb {T}]$, we define the auxiliary estimator

4.15 $$\begin{aligned} \widetilde{\rho }^{\,2}_\star :=\sum _{T\in \mathcal {T}} \widetilde{\rho }^{\,2}_{\star }(T) \quad \text {with}\ \widetilde{\rho }^{\,2}_{\star }(T):=\Vert \widetilde{h}_\star ^{1/2} \partial _{\varGamma } (f-V{\varPhi }_\star )\Vert _{L^2(T)}^2 \end{aligned}$$

which employs the novel mesh-size function $\widetilde{h}_\star$ from Proposition 4.2. Obviously the estimators $\mu _\star$ and $\widetilde{\rho }_\star$ are locally equivalent

4.16 $$\begin{aligned} {\widetilde{\rho }_\star ^{\,2}(T) \lesssim \mu _\star ^2(z)\lesssim \sum _{\begin{array}{c} {T'\in \mathcal {T}_\star }\\ {z\in T'} \end{array}} {\widetilde{\rho }}_\star ^{\,2}(T') \quad \text {for all }z\in \mathcal {N}_\star \text { and }T\in \mathcal {T}_\star \text { with }z\in T,} \end{aligned}$$

where the hidden constants depend only on $\check{\kappa }_{\max }$, *p*, and $\gamma$. The proof of the following lemma is inspired by [26, Proposition 3.2] resp. [8, Lemma 8.8], where only *h*-refinement is considered.

### Lemma 4.4

(Estimator reduction of $\widetilde{\rho })$ Algorithm 3.1 guarantees

4.17 $$\begin{aligned} \widetilde{\rho }_{\ell +1}^{\,2} \le q_\mathrm{est}\widetilde{\rho }_\ell ^{\,2} +C_\mathrm{est}\Vert {\varPhi }_{\ell +1}-{\varPhi }_\ell \Vert _{\widetilde{H}^{-1/2}({\varGamma })}^2\quad \text {for all }\ell \ge 0. \end{aligned}$$

The constants $0<q_\mathrm{est}<1$ and $C_\mathrm{est}>0$ depend only on $\check{\kappa }_{\max },p,w_{\min },w_{\max },\gamma ,$ and $\theta$.

### Proof

The proof is done in several steps.

*Step 1* With the inverse estimate (4.2), there holds the following stability property for any measurable ${\varGamma }_0\subseteq {\varGamma }$

$$\begin{aligned}&\left| \Vert \widetilde{h}_{\ell +1}^{1/2} \partial _{\varGamma } (f-V{\varPhi }_{\ell +1})\Vert _{L^2({\varGamma }_0)}-\Vert \widetilde{h}_{\ell +1}^{1/2} \partial _{\varGamma } (f-V{\varPhi }_{\ell })\Vert _{L^2({\varGamma }_0)}\right| \\&\quad \le \Vert \widetilde{h}_{\ell +1}^{1/2} \partial _{\varGamma } V({\varPhi }_{\ell +1}-{\varPhi }_{\ell })\Vert _{L^2({\varGamma }_0)}\le C \Vert {\varPhi }_{\ell +1}-{\varPhi }_\ell \Vert _{\widetilde{H}^{-1/2}({\varGamma })}, \end{aligned}$$

with a constant $C>0$ which depends only on $C_\mathrm{wt},C_\mathrm{inv}$, and $\gamma$.

*Step 2* With Proposition 4.2, we split the estimator into a contracting and into a non-contracting part

$$\begin{aligned} \widetilde{\rho }_{\ell +1}^{\,2}= & {} \Vert \widetilde{h}_{\ell +1}^{1/2} \partial _{\varGamma }(f-V{\varPhi }_{\ell +1})\Vert _{L^2(\omega _{\ell +1}([\mathcal {T}_{\ell +1}] {\setminus }[\mathcal {T}_{\ell }]))}^2\\&+\,\Vert \widetilde{h}^{1/2}_{\ell +1} \partial _{\varGamma }(f-V{\varPhi }_{\ell +1})\Vert _{L^2({\varGamma }{\setminus } \omega _{\ell +1}([\mathcal {T}_{\ell +1}]{\setminus }[\mathcal {T}_{\ell }]))} \end{aligned}$$

Step 1, the Young inequality, and Proposition 4.2 show, for arbitrary $\delta >0$, that

$$\begin{aligned}&\Vert \widetilde{h}_{\ell +1}^{1/2}\partial _{\varGamma } (f-V{\varPhi }_{\ell +1})\Vert _{L^2(\omega _{{\ell +1}}([\mathcal {T}_{\ell +1}]{\setminus }[\mathcal {T}_{\ell }]))}^2\\&\quad \le (1+\delta ) \Vert \widetilde{h}_{\ell +1}^{1/2} \partial _{\varGamma }(f-V{\varPhi }_{\ell })\Vert _{L^2(\omega _{{\ell +1}}([\mathcal {T}_{\ell +1}]{\setminus }[\mathcal {T}_{\ell }]))}^2\\&\qquad +\,(1+\delta ^{-1}) C^2 \Vert {\varPhi }_{\ell +1}-{\varPhi }_\ell \Vert _{\widetilde{H}^{-1/2}({\varGamma })}^2\\&\quad \le (1+\delta ) q_\mathrm{ctr}\Vert \widetilde{h}_{\ell }^{1/2} \partial _{\varGamma }(f-V{\varPhi }_\ell )\Vert _{L^2(\omega _{{\ell }}([\mathcal {T}_{\ell }]{\setminus }[\mathcal {T}_{\ell +1}]))}^2\\&\qquad +\,(1+\delta ^{-1}) C^2\Vert {\varPhi }_{\ell +1}-{\varPhi }_\ell \Vert _{\widetilde{H}^{-1/2}({\varGamma })}^2. \end{aligned}$$

Analogously, we get

$$\begin{aligned}&\Vert \widetilde{h}_{\ell +1}^{1/2}\partial _{\varGamma } (f-V{\varPhi }_{\ell +1})\Vert _{L^2({\varGamma }{\setminus }\omega _{{\ell +1}} ([\mathcal {T}_{\ell +1}]{\setminus }[\mathcal {T}_{\ell }]))}^2\\&\quad \le (1+\delta ) \Vert \widetilde{h}_{\ell }^{1/2} \partial _{\varGamma }(f-V{\varPhi }_\ell )\Vert _{L^2({\varGamma }{\setminus } \omega _{{\ell }}([\mathcal {T}_{\ell }]{\setminus }[\mathcal {T}_{\ell +1}]))}^2\\&\qquad +\,(1+\delta ^{-1}) C^2 \Vert {\varPhi }_{\ell +1}-{\varPhi }_\ell \Vert _{\widetilde{H}^{-1/2}({\varGamma })}^2. \end{aligned}$$

Combining these estimates, we end up with

4.18 $$\begin{aligned} \widetilde{\rho }_{\ell +1}^{\,2}\le & {} (1+\delta )\widetilde{\rho }_\ell ^{\,2}-(1+\delta )(1- q_\mathrm{ctr})\Vert \widetilde{h}_{\ell }^{1/2}\partial _{\varGamma }(f-V{\varPhi }_\ell )\Vert _{L^2(\omega _{{\ell }} ([\mathcal {T}_{\ell }]{\setminus }[\mathcal {T}_{\ell +1}]))}^2\nonumber \\&+\,2(1+\delta ^{-1}) C^2 \Vert {\varPhi }_{\ell +1}-{\varPhi }_\ell \Vert _{\widetilde{H}^{-1/2}({\varGamma })}^2. \end{aligned}$$

*Step 3* Local equivalence (4.16) and the Dörfler marking (3.3) for $\mu _\ell$ imply

$$\begin{aligned} \theta \widetilde{\rho }_\ell ^{\,2}\simeq \theta \mu _\ell ^2 \le \sum _{z\in \mathcal M_\ell }\mu _\ell (z)^2\simeq \sum _{\begin{array}{c} T\in \mathcal {T}_\ell \\ T\subseteq \omega _{{\ell }}(\mathcal M_\ell ) \end{array}} \widetilde{\rho }_\ell (T)^2, \end{aligned}$$

where the hidden constants depend only on $\check{\kappa }_\mathrm{max}$, *p*, and $\gamma$. Hence, $\widetilde{\rho }_\ell$ satisfies some Dörfler marking with a certain parameter $0<\widetilde{\theta }<1$. With $\mathcal M_\ell \subseteq \bigcup ([\mathcal {T}_\ell ]{\setminus } [\mathcal {T}_{\ell +1}])$, (4.18) hence becomes

$$\begin{aligned} \widetilde{\rho }_{\ell +1}^{\,2}&\le \left( (1+\delta )-(1+\delta )(1- q_\mathrm{ctr}){\widetilde{\theta }}\right) {\widetilde{\rho }}_\ell ^{\,2} +2(1+\delta ^{-1}) C^2 \Vert {\varPhi }_{\ell +1}-{\varPhi }_\ell \Vert _{\widetilde{H}^{-1/2}({\varGamma })}^2. \end{aligned}$$

By choosing $\delta$ sufficiently small, we prove (4.17) with $C_\mathrm{est}:=2(1+\delta ^{-1})C^2$ and $q_\mathrm{est}:=(1+\delta )\big (1-(1-q_\mathrm{ctr})\widetilde{\theta }\big )<1$. $\square$

### Proof of linear convergence (3.5)

Due to the properties of the weakly-singular integral operator *V*, the bilinear form $A(\phi ,\psi ):=\langle V\phi \,;\,\psi \rangle _{{\varGamma }}$ defines even a scalar product, and the induced norm $\Vert \psi \Vert _{V}:=A(\psi ,\psi )^{1/2}$ is an equivalent norm on $\widetilde{H}^{-1/2}({\varGamma })$. According to nestedness of the ansatz spaces $\mathcal {X}_{\ell }\subset \mathcal {X}_{\ell +1}$, the Galerkin orthogonality implies the Pythagoras theorem

$$\begin{aligned} \Vert \phi -{{\varPhi }}_{\ell +1}\Vert _{V}^2 + \Vert {{\varPhi }}_{\ell +1}-{{\varPhi }}_\ell \Vert _{V}^2 = \Vert \phi -{{\varPhi }}_\ell \Vert _{V}^2 \quad \text {for all }\ell \in \mathbb {N}_0. \end{aligned}$$

Together with the estimator reduction (4.17) and reliability (3.2)

$$\begin{aligned} \Vert \phi -{\varPhi }_\ell \Vert _{V} \simeq \Vert \phi -{\varPhi }_\ell \Vert _{\widetilde{H}^{-1/2}({\varGamma })} \lesssim \mu _\ell \simeq {\widetilde{\rho }}_\ell , \end{aligned}$$

this implies the existence of $0<\kappa ,\lambda <1$, which depend only on $C_\mathrm{rel},C_\mathrm{est}$ and $q_\mathrm{est}$, such that ${\varDelta }_\star :=\Vert \phi -{\varPhi }_\star \Vert _{V}^2+\lambda \,{\widetilde{\rho }}_\star ^{\,2} \simeq {\widetilde{\rho }}_\star ^{\,2}$ satisfies

$$\begin{aligned} {\varDelta }_{\ell +1} \le \kappa \,{\varDelta }_\ell \quad \text {for all }\ell \in \mathbb {N}_0; \end{aligned}$$

see, e.g., [26, Theorem 4.1], while the original idea goes back to [10]. From this, we infer

$$\begin{aligned} \mu _{\ell +n}^2\simeq \,{\widetilde{\rho }}_{\ell +n}^{\,2} \simeq {\varDelta }_{\ell +n} \le \kappa ^n\,{\varDelta }_\ell \simeq \kappa ^n\,{\widetilde{\rho }}_{\ell }^{\,2} \simeq \kappa ^n\,\mu _{\ell }^2 \quad \text {for all }\ell ,n\in \mathbb {N}_0 \end{aligned}$$

and hence conclude the proof. $\square$

## Proof of Theorem 3.2, optimal convergence (3.7)

As in the previous section, we define an auxiliary error estimator. For each $[\mathcal {T}_\star ]\in [\mathbb {T}]$, let

5.1 $$\begin{aligned} {\rho }^2_\star :=\sum _{T\in \mathcal {T}} {\rho }_{\star }(T)^2 \quad \text {with }{\rho }_{\star }(T)^2:=\Vert \check{h}_\star ^{1/2}\partial _{\varGamma } (f-V{\varPhi }_\star )\Vert _{L^2(T)}^2. \end{aligned}$$

Note that the estimators $\mu _\star$ and $\rho _\star$ are again locally equivalent

5.2 $$\begin{aligned} {\rho _\star ^{\,2}(T) \le \mu _\star ^{\,2}(z)\lesssim \sum _{\begin{array}{c} {T'\in \mathcal {T}_\star } \\ {z\in T'} \end{array}}\rho _\star ^{\,2}(T')\quad \text {for all }z\in \mathcal {N}_\star \text { and } T\in \mathcal {T}_\star \text { with } z\in T,} \end{aligned}$$

where the hidden constant depends only on $\check{\kappa }_{\max }$. Analogous versions of the next two lemmas are already proved in [26, Proposition 4.2 and 4.3] for *h*-refinement and piecewise constants; see also [8, Propostion 5.7] for discontinuous piecewise polynomials and *h*-refinement. The proof for Lemma 5.1 is essentially based on Proposition 4.1. The proof of Lemma 5.2 requires the construction of a Scott–Zhang type operator (5.9) which is not necessary in [8, 26], since both works consider discontinuous piecewise polynomials.

### Lemma 5.1

(Stability of $\rho$) Let $[\mathcal {T}_\star ]\in [\mathbb {T}]$ and $[\mathcal {T}_+]\in \mathtt{ref}(\mathcal {T}_\star )$. For $\mathcal {S}\subseteq \mathcal {T}_\star \cap \mathcal {T}_+$ there holds

5.3 $$\begin{aligned} \left| \left( \sum _{T\in \mathcal {S}} \rho _{+}(T)^2\right) ^{1/2}-\left( \sum _{T\in \mathcal {S}} \rho _\star (T)^2\right) ^{1/2}\right| \le C_\mathrm{stab}\Vert {\varPhi }_{+}-{\varPhi }_\star \Vert _{\widetilde{H}^{-1/2}({\varGamma })}, \end{aligned}$$

where $C_\mathrm{stab}>0$ depends only on the parametrization $\gamma$ and the constant $C_\mathrm{inv}$ of Proposition 4.1.

### Proof

For all subsets ${\varGamma }_0\subseteq {\varGamma }$, it holds

5.4 $$\begin{aligned}&\left| \Vert \check{h}_{+}^{1/2}\partial _{\varGamma } (f-V{\varPhi }_{+})\Vert _{L^2({\varGamma }_0)} -\Vert \check{h}_{+}^{1/2}\partial _{\varGamma } (f-V{\varPhi }_\star )\Vert _{L^2({\varGamma }_0)}\right| \nonumber \\&\quad \le \Vert \check{h}_{+}^{1/2}\partial _{\varGamma } V({\varPhi }_{+}-{\varPhi }_\star )\Vert _{L^2({\varGamma }_0)} \nonumber \\&\quad \lesssim \Vert {h}_{+}^{1/2}\partial _{\varGamma } V({\varPhi }_{+}-{\varPhi }_\star )\Vert _{L^2({\varGamma }_0)} \le C_\mathrm{inv}\Vert {\varPhi }_{+}-{\varPhi }_\star \Vert _{\widetilde{H}^{-1/2}({\varGamma })}. \end{aligned}$$

The choice ${\varGamma }_0=\bigcup \mathcal {S}$ shows stability

$$\begin{aligned} \left| \left( \sum _{T\in \mathcal {S}} \rho _{+}(T)^2\right) ^{1/2}-\left( \sum _{T\in \mathcal {S}} \rho _\star (T)^2\right) ^{1/2}\right| \le C_\mathrm{inv}\Vert {\varPhi }_{+}-{\varPhi }_\star \Vert _{\widetilde{H}^{-1/2}({\varGamma })}, \end{aligned}$$

and we conclude the proof. $\square$

### Lemma 5.2

(Discrete reliability of $\rho )$ There exist constants $C_\mathrm{rel}, C_\mathrm{ref}>0,$ which depend only on $\check{\kappa }_{\max }, p, w_{\min }, w_{\max },$ and $\gamma ,$ such that for all refinements $[\mathcal {T}_{+}]\in \mathtt{ref}([\mathcal {T}_\star ])$ of $[\mathcal {T}_\star ]\in [\mathbb {T}]$ there exists a subset $\mathcal {R}_\star (\mathcal {T}_+)\subseteq \mathcal {T}_\star$ with

5.5 $$\begin{aligned} \Vert {\varPhi }_{+}-{\varPhi }_\star \Vert _{\widetilde{H}^{-1/2}({\varGamma })}^2\le C_\mathrm{rel}\sum _{T\in \mathcal {R}_\star (\mathcal {T}_+)} \rho _\star (T)^2 \end{aligned}$$

as well as

5.6 $$\begin{aligned} {\bigcup ([\mathcal {T}_\star ]{\setminus }[\mathcal {T}_{+}])\subseteq \bigcup \mathcal {R}_\star (\mathcal {T}_{+})}\quad \text {and}\quad |\mathcal {R}_\star (\mathcal {T}_+)|\le C_\mathrm{ref}|[\mathcal {T}_\star ]{\setminus }[\mathcal {T}_{+}]|. \end{aligned}$$

For the proof of Lemma 5.2, we need to introduce a Scott–Zhang type operator. Let $[\mathcal {T}_\star ]\in [\mathbb {T}]$ and $\big \{R_{i,p}|_{[a,b\rangle }\,:\,i=1-p,\dots ,N-p\big \}\circ \gamma |_{[a,b\rangle }^{-1}$ be the basis of NURBS of $\mathcal {X}_\star$, where “$\rangle$” stands for “)” if ${\varGamma }=\partial {\varOmega }$ is closed and for “]” if ${\varGamma }\subsetneqq \partial {\varOmega }$ is open. Here, *N* denotes the number of transformed knots $\check{\mathcal {K}}_\star$ in (*a*, *b*]. With the corresponding B-splines there holds $R_{i,p}=w_iB_{i,p} /w$, where $w=\sum _{\ell \in \mathbb {Z}} w_\ell B_{\ell ,p}$ is the fixed denominator satisfying $w_\mathrm{min}\le w\le w_\mathrm{max}$; see Sect. 2.8. In [4, Section 2.1.5], it is shown that, for $i\in \{1-p,\dots ,N-p\}$, there exist dual basis functions $B_{i,p}^*\in L^2(\mathrm{supp}B_{i,p})$ with

5.7 $$\begin{aligned} \int _{\mathrm{supp}B_{i,p}} B_{i,p}^*(t) B_{j,p}(t) dt=\delta _{ij}= {\left\{ \begin{array}{ll} 1&{} \text {if }i=j,\\ 0&{}\text {else}, \end{array}\right. } \end{aligned}$$

and

5.8 $$\begin{aligned} \Vert B_{i,p}^*\Vert _{L^2(\mathrm{supp}B_{i,p})}\le (2p+3)9^p {|\mathrm{supp}B_{i,p}|^{-1/2}}. \end{aligned}$$

Define $R_{i,p}^*:=B_{i,p}^* w/w_i$ with the denominator *w* from before, and $\widehat{R}_{i,p}:=R_{i,p}|_{[a,b\rangle }\circ \gamma |_{[a,b\rangle }^{-1}$. For $I\subseteq \{1-p,\dots ,N-p\}$, we define the following Scott–Zhang type operator

5.9 $$\begin{aligned} P_{\star ,I}:L^2({\varGamma })\rightarrow \mathcal {X}_\star :\psi \mapsto \sum _{i\in I} \left( \int _{\mathrm{supp}{R}_{i,p}} R_{i,p}^*(t) \psi (\gamma (t)) \,dt \right) \widehat{R}_{i,p}. \end{aligned}$$

In [4, Section 3.1.2], a similar operator is considered for $I= \{1-p,\dots ,N-p\}$, and [4, Proposition 2.2] proves an analogous version of the following lemma.

### Lemma 5.3

The Scott–Zhang type operator (5.9) satisfies the following two properties : 

(i) Local projection property :  For $T\in \mathcal {T}_\star$ with $\big \{i\,:\,T\subseteq \mathrm{supp}\widehat{R}_{i,p}\big \}\subseteq I$ and $\psi \in L^2({\varGamma }),$ the inclusion $\psi |_{\omega _\star ^{p}(T)} \in \mathcal {X}_\star |_{\omega _\star ^p(T)}:=\big \{\xi |_{\omega _\star ^p(T)}\,:\,\xi \in \mathcal {X}_\star \big \}$ implies $\psi |_T=(P_{\star ,I}\psi )|_T$.
(ii) Local $L^2$-stability :  For $\psi \in L^2({\varGamma })$ and $T\in \mathcal {T}_\star ,$ there holds
  $$\begin{aligned} \Vert P_{\star ,I}(\psi )\Vert _{L^2(T)}\le C_\mathrm{sz}\Vert \psi \Vert _{L^2(\omega _\star ^{p}(T))}, \end{aligned}$$
  where $C_\mathrm{sz}$ depends only on $\check{\kappa }_{\max }, p, w_{\max }$, and $\gamma$.

### Proof

All NURBS basis functions which are non-zero on *T*, have support in $\omega _\star ^p(T)$. With this, (i) follows easily from the definition of $P_{\star ,I}$. For stability (ii), we use $0\le \widehat{R}_{i,p}\le 1$ and (5.8) to see

$$\begin{aligned} \Vert P_{\star ,I}\psi \Vert _{L^2(T)}&=\left\| \sum _{i\in I} \left( \int _{\mathrm{supp}{R}_{i,p}} {R}_{i,p}^*(t) \psi (\gamma (t)) \,dt\right) \widehat{R}_{i,p}\right\| _{L^2(T)}\\&\le \sum _{\begin{array}{c} {i\in I} \\ {|\mathrm{supp}\widehat{R}_{i,p}\cap T|>0} \end{array}} \left| \int _{\mathrm{supp}{R}_{i,p}} {R}_{i,p}^*(t) \psi (\gamma (t))\, dt\right| \Vert \widehat{R}_{i,p}\Vert _{L^2(T)}\\&\lesssim \sum _{\begin{array}{c} {i\in I} \\ {|\mathrm{supp}\widehat{R}_{i,p}\cap T|>0} \end{array}} \Vert {R}_{i,p}^*\Vert _{L^2(\mathrm{supp}{R}_{i,p})} {\Vert \psi \Vert _{L^2(\mathrm{supp}\widehat{R}_{i,p})}}h_{\star ,T}^{1/2}\\&\mathop {\lesssim }\limits ^{(5.8)}\sum _{\begin{array}{c} {i\in I} \\ {|\mathrm{supp}\widehat{R}_{i,p}\cap T|>0} \end{array}} \Vert \psi \Vert _{L^2(\mathrm{supp}\widehat{R}_{i,p})}\lesssim \Vert \psi \Vert _{L^2(\omega _\star ^p(T))}. \end{aligned}$$

Overall, the hidden constants depend only on $\check{\kappa }_{\max }, p, w_{\max }$, and $\gamma$. $\square$

### Proof of Lemma 5.2

We choose

$$\begin{aligned} I:=\big \{i\,:\,|\mathrm{supp}\widehat{R}_{i,p}\cap {\varGamma }{\setminus } \omega _\star ^{p}([\mathcal {T}_\star ]{\setminus } [\mathcal {T}_{+}])|>0\big \}. \end{aligned}$$

We prove that

5.10 $$\begin{aligned} P_{\star ,I}({\varPhi }_{+}-{\varPhi }_\star )= {\left\{ \begin{array}{ll} {\varPhi }_{+}-{\varPhi }_\star &{}\text { on } {\varGamma }{\setminus }\omega _\star ^{p}( [\mathcal {T}_\star ]{\setminus } [\mathcal {T}_{+}]),\\ 0&{} \text{ on } \bigcup ([\mathcal {T}_\star ]{\setminus } [\mathcal {T}_{+}]). \end{array}\right. } \end{aligned}$$

To see this, let $T\in \mathcal {T}_\star$ with $T\subseteq \overline{{\varGamma }{\setminus }\omega _\star ^{p}( [\mathcal {T}_\star ]{\setminus } [\mathcal {T}_{+}])}$. Then, $\big \{i\,:\,T\subseteq \mathrm{supp}\widehat{R}_{i,p}\big \}\subseteq I$. It holds $\omega _\star ^{p}(T)\subseteq \bigcup ([\mathcal {T}_\star ]\cap [\mathcal {T}_{+}])$. This implies that no new knots are inserted in $\omega ^p_\star (T)$. With Lemma 2.3(i), it follows $\mathcal {X}_+|_{\omega _\star ^p(T)}=\mathcal {X}_\star |_{\omega _\star ^p(T)}$ and, in particular, $({\varPhi }_{+}-{\varPhi }_\star )|_{\omega _\star ^p(T)}\in \mathcal {X}_\star |_{\omega ^p_\star (T)}$. Hence Lemma 5.3(i) is applicable and proves $P_{\star ,I}({\varPhi }_+-{\varPhi }_\star )|_T=({\varPhi }_+-{\varPhi }_\star )|_T$. For $T\in \mathcal {T}_\star$ with $T\subseteq \bigcup ([\mathcal {T}_\star ]{\setminus } [\mathcal {T}_+])$, the assertion follows immediately from the definition of $P_{\star ,I}$, since $\widehat{R}_{i,p}|_T=0$ for $i\in I$.

Let $\widetilde{\mathcal {N}}_\star :=\big \{z\in \mathcal {N}_\star \,:\,z\in \omega _\star ^{p}( [\mathcal {T}_\star ]{\setminus } [\mathcal {T}_{+}])\big \}$. For $z\in \widetilde{\mathcal {N}}_\star$, let $\varphi _z$ be the $P^1$ hat function, i.e., $\varphi _z(z')=\delta _{zz'}$ for all $z'\in \mathcal {N}_\star$, $\mathrm{supp}(\varphi _z)=\omega _\star (z)$, and $\partial _{\varGamma } \varphi _z= \mathrm {const.}$ on $T_{z,1}$ and $T_{z,2}$, where $\omega _\star (z)=T_{z,1}\cup T_{z,2}$ with $T_{z,1},T_{z,2}\in \mathcal {T}_\star$. Because of Galerkin orthogonality and $\sum _{z\in \widetilde{\mathcal {N}}_\star } \varphi _z=1$ on $\omega _\star ^{p}( [\mathcal {T}_\star ]{\setminus } [\mathcal {T}_{+}])$, we see

$$\begin{aligned} \Vert {\varPhi }_{+}-{\varPhi }_\star \Vert _{V}^2= & {} \langle f-V{\varPhi }_\star \,;\,(1-P_{\star ,I}) ({\varPhi }_+-{\varPhi }_\star )\rangle _{\varGamma }\\= & {} \left\langle \sum _{z\in \widetilde{\mathcal {N}}_\star } \varphi _z (f-V{\varPhi }_\star );(1-P_{\star ,I})({\varPhi }_{+}-{\varPhi }_\star )\right\rangle _{\varGamma }. \end{aligned}$$

We abbreviate ${\varSigma }:=\sum _{z\in \widetilde{\mathcal {N}}} \varphi _z (f-V{\varPhi }_\star )$ and estimate with (M1), Lemma 5.3(ii) and Proposition 4.1

$$\begin{aligned}&\langle {\varSigma }\,;\,P_{\star ,I}({\varPhi }_{+}-{\varPhi }_\star )\rangle \le \Vert h_\star ^{-1/2}{\varSigma }\Vert _{L^2({\varGamma })}\Vert h_\star ^{1/2} P_{\star ,I}({\varPhi }_{+}-{\varPhi }_\star )\Vert _{L^2({\varGamma })}\\&\quad \mathop {=}\limits ^{(5.10)}\Vert h_\star ^{-1/2} {\varSigma }\Vert _{L^2({\varGamma })} \Vert h_{+}^{1/2} P_{\star ,I}({\varPhi }_{+}-{\varPhi }_\star )\Vert _{L^2(\bigcup ([\mathcal {T}_\star ]\cap [\mathcal {T}_{+}]))} \\&\quad \mathop {\lesssim }\limits ^{\text {Lem.}~5.3} \Vert h_\star ^{-1/2} {\varSigma }\Vert _{L^2({\varGamma })} \Vert h_{+}^{1/2} ({\varPhi }_{+}-{\varPhi }_\star )\Vert _{L^2(\omega _\star ^p( [\mathcal {T}_\star ]\cap [\mathcal {T}_{+}]))}\\&\quad \mathop {\lesssim }\limits ^{\text {Prop.}~4.1} \Vert h_\star ^{-1/2} {\varSigma }\Vert _{L^2({\varGamma })} \Vert {\varPhi }_{+}-{\varPhi }_\star \Vert _{V}, \end{aligned}$$

as well as

$$\begin{aligned} \langle {\varSigma }\,;\,{\varPhi }_{+}-{\varPhi }_\star \rangle \le \Vert {\varSigma }\Vert _{H^{1/2}({\varGamma })} \Vert {\varPhi }_{+}-{\varPhi }_\star \Vert _{\widetilde{H}^{-1/2}({\varGamma })}\simeq \Vert {\varSigma }\Vert _{H^{1/2}({\varGamma })}\Vert {\varPhi }_{+}-{\varPhi }_\star \Vert _{V}. \end{aligned}$$

So far, we thus have proved

5.11 $$\begin{aligned} \Vert {\varPhi }_{+}-{\varPhi }_\star \Vert _{V}\le & {} \Vert h_\star ^{-1/2}{\varSigma }\Vert _{L^2({\varGamma })} +\Vert {\varSigma }\Vert _{H^{1/2}({\varGamma })}\nonumber \\\le & {} \Vert h_\star ^{-1/2}(f-V{\varPhi }_\star )\Vert _{L^2(\omega ^{p+1}([\mathcal {T}_\star ]{\setminus } [\mathcal {T}_{+}]))}+\Vert {\varSigma }\Vert _{H^{1/2}({\varGamma })}. \end{aligned}$$

Next, we use [25, Lemma 3.4], [24, Lemma 4.5], and $|\partial _{\varGamma }\varphi _z|\simeq |\omega _\star (z)|^{-1}$ to estimate

5.12 $$\begin{aligned}&\Vert {\varSigma }\Vert _{H^{1/2}({\varGamma })}^2 \mathop {\lesssim }\limits ^{[25]}\sum _{z\in \mathcal {N}_\star } |{\varSigma }|^2_{H^{1/2}(\omega _\star (z))}+\Vert h_\star ^{-1/2}{\varSigma }\Vert _{L^2({\varGamma })}^2\nonumber \\&\quad \mathop {\lesssim }\limits ^{[24]}\sum _{z\in \mathcal {N}_\star }\Vert h_\star ^{1/2} \partial _{\varGamma } {\varSigma }\Vert _{L^2(\omega _\star (z))}^2+\Vert h_\star ^{-1/2}{\varSigma }\Vert _{L^2({\varGamma })}^2\nonumber \\&\quad \lesssim \Vert h_\star ^{1/2}\partial _{\varGamma } {\varSigma }\Vert _{L^2({\varGamma })}^2+\Vert h_\star ^{-1/2}{\varSigma }\Vert _{L^2({\varGamma })}^2\nonumber \\&\quad \lesssim \left\| {h_\star ^{1/2} \partial _{\varGamma }(f-V{\varPhi }_\star )\sum _{z\in \widetilde{\mathcal {N}}_\star } \varphi _z}\right\| _{L^2({\varGamma })}^2+\left\| {h_\star ^{1/2}(f-V{\varPhi }_\star ) \sum _{z\in \widetilde{\mathcal {N}}_\star } \partial _{\varGamma } \varphi _z }\right\| _{L^2({\varGamma })}^2\nonumber \\&\qquad +\,\Vert h_\star ^{-1/2}{\varSigma }\Vert _{L^2({\varGamma })}^2\nonumber \\&\quad \lesssim \Vert h_\star ^{1/2} \partial _{\varGamma }(f- V{\varPhi }_\star )\Vert _{L^2(\omega _\star ^{p+1}([\mathcal {T}_\star ]{\setminus } [\mathcal {T}_{+}]))}^2+ \Vert h_\star ^{-1/2}(f-V{\varPhi }_\star )\Vert _{L^2(\omega _\star ^{p+1}([\mathcal {T}_\star ]{\setminus } [\mathcal {T}_{+}]))}^2.\nonumber \\ \end{aligned}$$

It remains to consider the term $\Vert h_\star ^{-1/2}(f-V{\varPhi }_\star )\Vert _{L^2(\omega ^{p+1}([\mathcal {T}_\star ]{\setminus } [\mathcal {T}_{+}]))}$ of (5.11) and (5.12). It holds

5.13 $$\begin{aligned} \Vert h_\star ^{-1/2} (f-V{\varPhi }_\star )\Vert _{L^2(\omega _\star ^{p+1}([\mathcal {T}_\star ]{\setminus } [\mathcal {T}_{+}]))}^2=\sum _{\begin{array}{c} {T\in \mathcal {T}_\star } \\ {T\subseteq \omega _\star ^{p+1}([\mathcal {T}_\star ]{\setminus } [\mathcal {T}_{+}])} \end{array}}\Vert h_\star ^{-1/2}(f-V{\varPhi }_\star )\Vert _{L^2(T)}^2.\nonumber \\ \end{aligned}$$

For any $T\in \mathcal {T}_\star$, there is a function $\psi _T\in \mathcal {X}_\star$ with connected support, $T\subseteq \mathrm{supp}(\psi _T)\subseteq \omega _\star ^{\lceil p/2\rceil }(T)$ and $\Vert 1-\psi _T\Vert _{L^2(\mathrm{supp}(\psi _T))}^2\le q\, |\mathrm{supp}(\psi _T)|$ with some $q\,\in (0,1)$ which depends only on $\check{\kappa }_\mathrm{max}, \gamma , p, w_\mathrm{min}$, and $w_\mathrm{max}$; see [25, (A1)–(A2), Theorem 4.4]. We use some Poincaré inequality (see, e.g., [18, Lemma 2.5]) to see

5.14 $$\begin{aligned} \Vert f-V{\varPhi }_\star \Vert _{L^2(\mathrm{supp}{\psi }_T)}^2\le & {} {\frac{|\mathrm{supp}(\psi _T)|^2}{2}} \Vert \partial _{\varGamma } (f-V{\varPhi }_\star )\Vert _{L^2(\mathrm{supp}(\psi _T))}^2\nonumber \\&+\,\frac{1}{|\mathrm{supp}(\psi _T)|} \left| \int _{\mathrm{supp}(\psi _T)} (f-V{\varPhi }_\star )(x) \,dx\right| ^2.\quad \quad \quad \end{aligned}$$

The Galerkin orthogonality proves

$$\begin{aligned} \left| \int _{\mathrm{supp}(\psi _T)} (f-V{\varPhi }_\star )(x) \,dx\right| ^2= & {} \left| \int _{\mathrm{supp}(\psi _T)} (f-V{\varPhi }_\star )(x)(1-\psi _T(x))\,dx\right| ^2\\\le & {} \Vert f-V{\varPhi }_\star \Vert _{L^2(\mathrm{supp}(\psi _T))}^2 q\, |\mathrm{supp}(\psi _T)|. \end{aligned}$$

Using (5.14), we therefore get

$$\begin{aligned} \Vert f-V{\varPhi }_\star \Vert _{L^2(\mathrm{supp}(\psi _T))}^2\le & {} \frac{|\mathrm{supp}(\psi _T)|^2}{2} \Vert \partial _{\varGamma }(f-V{\varPhi }_\star )\Vert _{L^2(\mathrm{supp}(\psi _T))}^2\\&+\,q\,\Vert f-V{\varPhi }_\star \Vert _{L^2(\mathrm{supp}(\psi _T))}^2, \end{aligned}$$

which implies

$$\begin{aligned} \Vert f-V{\varPhi }_\star \Vert _{L^2(T)}^2\lesssim {h_{\star ,T}^2}\Vert \partial _{\varGamma }(f-V{\varPhi }_\star )\Vert _{L^2(\omega ^{\lceil p/2 \rceil }(T))}^2. \end{aligned}$$

Hence, we are led to

$$\begin{aligned} \Vert h_\star ^{-1/2} (f-V{\varPhi }_\star )\Vert _{L^2(\omega ^{p+1}([\mathcal {T}_\star ]{\setminus } [\mathcal {T}_{+}]))}^2\lesssim \Vert h_\star ^{1/2} \partial _{\varGamma }(f-V{\varPhi }_\star )\Vert _{L^2(\omega ^{\lceil p/2\rceil + p+1}([\mathcal {T}_\star ]{\setminus } [\mathcal {T}_{+}]))}^2. \end{aligned}$$

With

$$\begin{aligned} \mathcal {R}_\star (\mathcal {T}_{+}):=\big \{T\in \mathcal {T}_\star \,:\,T\subseteq \omega ^{\lceil p/2\rceil +p+1}([\mathcal {T}_\star ]{\setminus } [\mathcal {T}_{+}])\big \}, \end{aligned}$$

we therefore conclude the proof. $\square$

Since we use a different mesh-refinement strategy, we cannot directly cite the following lemma from [8]. However, we may essentially follow the proof of [8, Proposition 4.12] verbatim. Details are left to the reader.

### Lemma 5.4

(Optimality of Dörfler marking) Define

5.15 $$\begin{aligned} \overline{\theta }_\mathrm{opt}:=(1+C_\mathrm{stab}^2C_\mathrm{rel}^2)^{-1}. \end{aligned}$$

For all $0<\overline{\theta }<\overline{\theta }_\mathrm{opt}$ there is some $0<q_\mathrm{opt}<1$ such that for all refinements $[\mathcal {T}_{+}]\in \mathtt{ref}([\mathcal {T}_\star ])$ of $[\mathcal {T}_\star ]\in [\mathbb {T}]$ the following implication holds true

5.16 $$\begin{aligned} \rho _{+}^2\le q_\mathrm{opt}\rho _\star ^2\quad \Longrightarrow \quad \overline{\theta }\rho _\star ^2\le \sum _{T\in \mathcal {R}_\star (\mathcal {T}_+)} \rho _\star (T)^2. \end{aligned}$$

The constant $q_\mathrm{opt}$ depends only on $\overline{\theta }$ and the constants $C_\mathrm{stab}$ of Lemma 5.1 and $C_\mathrm{rel}$ of Lemma 5.2. $\square$

The next lemma reads similarly as [8, Lemma 3.4]. Since we use a different mesh-refinement strategy and our estimator $\rho$ does not satisfy the reduction axiom (A2), we cannot directly cite the result. However, the idea of the proof is the same. Indeed, one only needs a weaker version of the mentioned axiom.

### Lemma 5.5

(Quasi-monotonicity of $\rho )$ For all refinements $[\mathcal {T}_{+}]\in \mathtt{ref}([\mathcal {T}_\star ])$ of $[\mathcal {T}_\star ]\in [\mathbb {T}],$ there holds

5.17 $$\begin{aligned} \rho _{+}^2\le C_\mathrm{mon}\rho _\star ^2, \end{aligned}$$

where $C_\mathrm{mon}>0$ depends only on the parametrisation $\gamma$ and the constants $C_\mathrm{inv}$ of Proposition 4.1 and $C_\mathrm{rel}$ of Lemma 5.2.

### Proof

We split the estimator as follows

$$\begin{aligned} \rho _{+}^2=\sum _{T\in \mathcal {T}_{+}{\setminus } \mathcal {T}_\star } \rho _{+}(T)^2+\sum _{T\in \mathcal {T}_\star \cap \mathcal {T}_{+}} \rho _+(T)^2. \end{aligned}$$

For the first sum, we use (5.4), $\bigcup (\mathcal {T}_{+}{\setminus } \mathcal {T}_\star )=\bigcup (\mathcal {T}_\star {\setminus } \mathcal {T}_{+})$, and $\check{h}_{+}\le \check{h}_\star$ to estimate

$$\begin{aligned} \sum _{T\in \mathcal {T}_{+}{\setminus } \mathcal {T}_\star }\rho _{+}(T)^2= & {} \Vert \check{h}_{+}^{1/2} \partial _{\varGamma } (f-V{\varPhi }_{+})\Vert _{L^2(\bigcup (\mathcal {T}_{+}{\setminus } \mathcal {T}_\star ))}^2\\\lesssim & {} \left( \Vert {\varPhi }_+-{\varPhi }_\star \Vert _{\widetilde{H}^{-1/2}({\varGamma })}+\Vert \check{h}_\star ^{1/2} \partial _{\varGamma } (f-V{\varPhi }_\star )\Vert _{L^2(\bigcup (\mathcal {T}_{\star }{\setminus } \mathcal {T}_+))}\right) ^2\\\le & {} 2 \Vert {\varPhi }_{+}-{\varPhi }_\star \Vert _{\widetilde{H}^{-1/2}({\varGamma })}^2 +{2}\sum _{T\in \mathcal {T}_\star {\setminus } \mathcal {T}_{+}} \rho _\star (T)^2 \end{aligned}$$

For the second sum, we use Lemma 5.1 to see

$$\begin{aligned} \sum _{T\in \mathcal {T}_\star \cap \mathcal {T}_{+}} \rho _{+}(T)^2\le 2\sum _{T\in \mathcal {T}_\star {\cap {\mathcal {T}}}_{+}} \rho _\star (T)^2+2C_\mathrm{stab}^2 \Vert {\varPhi }_\star -{\varPhi }_{+}\Vert _{\widetilde{H}^{-1/2}({\varGamma })}^2. \end{aligned}$$

We end up with

$$\begin{aligned} \rho _{+}^2\lesssim \Vert {\varPhi }_{+}-{\varPhi }_\star \Vert _{\widetilde{H}^{-1/2}({\varGamma })}^2 + {\rho _\star ^2}. \end{aligned}$$

Lemma 5.2 concludes the proof. $\square$

The optimality in Theorem 3.2 essentially follows from the following lemma. It was inspired by an analogous version from [8, Lemma 4.14].

### Lemma 5.6

Suppose that $\phi \in \mathbb {A}_s$ for some $s>0$. Then,  for all $0<\overline{\theta }<\overline{\theta }_\mathrm{opt}$ there exist constants $C_1,C_2>0$ such that for all meshes $[\mathcal {T}_\star ]\in [\mathbb {T}]$ there exists some refinement $[\mathcal {T}_{+}]\in \mathtt{ref}([\mathcal {T}_\star ])$ such that the corresponding set $\mathcal {R}_\star (\mathcal {T}_+)\subseteq \mathcal {T}_\star$ from Lemma 5.2 satisfies

5.18 $$\begin{aligned} |\mathcal {R}_\star (\mathcal {T}_+)|\le C_1C_2^{1/s}\Vert \phi \Vert _{\mathbb {A}_s}^{1/s}\rho _\star ^{-1/s}, \end{aligned}$$

and

5.19 $$\begin{aligned} \overline{\theta }\rho _\star ^2\le \sum _{T\in \mathcal {R}_\star (\mathcal {T}_+)}\rho _\star (T)^2. \end{aligned}$$

With the constants $C_\mathrm{rel}, C_\mathrm{mon},$ and $q_\mathrm{opt}$ from Lemmas 5.2, 5.4 and 5.5, it holds $C_1=2C_\mathrm{rel}$ and $C_2=(C_\mathrm{mon}q_\mathrm{opt}^{-1})^{1/2}$.

### Proof

We set $\alpha :=C_\mathrm{mon}^{-1} q_\mathrm{opt}$ with the constants of Lemma 5.4 and Lemma 5.5, and $\delta ^2:=\alpha \rho _\star ^2$.

*Step 1*  There exists $[\mathcal {T}_\delta ]\in [\mathbb {T}]$ with

$$\begin{aligned} \rho _\delta \le \delta \quad \text {and}\quad |\mathcal {K}_\delta |-|\mathcal {K}_0|\le \Vert \phi \Vert _{\mathbb {A}_s}^{1/s}\delta ^{-1/s}. \end{aligned}$$

Let $N\in \mathbb {N}_0$ be minimal with $(N+1)^{-s}\Vert \phi \Vert _{\mathbb {A}_s}\le \delta$. If $N=0$, then $\rho _0\le \Vert \phi \Vert _{\mathbb {A}_s}\le \delta$ and we can choose $[\mathcal {T}_\delta ]=[\mathcal {T}_0]$. If $N>0$, minimality of *N* implies $N^{-s}\Vert \phi \Vert _{\mathbb {A}_s}>\delta$ or equivalently $N< \Vert \phi \Vert _{\mathbb {A}_s}^{1/s}\delta ^{-1/s}$. We choose $[\mathcal {T}_\delta ]\in [\mathbb {T}_N]$ such that

$$\begin{aligned} \rho _\delta =\min _{[\mathcal {T}_\bullet ]\in [\mathbb {T}_N]} \rho _\bullet . \end{aligned}$$

By the definition of $\Vert \phi \Vert _{\mathbb {A}_s}$, we see

$$\begin{aligned} \rho _\delta \le (N+1)^{-s}\Vert \phi \Vert _{\mathbb {A}_s}\le \delta . \end{aligned}$$

By the definition of $[\mathbb {T}_N]$, we see

$$\begin{aligned} |\mathcal {K}_\delta |-|\mathcal {K}_0|\le N< \Vert \phi \Vert _{\mathbb {A}_s}^{1/s}\delta ^{-1/s}. \end{aligned}$$

*Step 2* We consider the overlay $[\mathcal {T}_{+}]:= [\mathcal {T}_\star ]\oplus [\mathcal {T}_\delta ]$ of (M2). Quasi-monotonicity shows

5.20 $$\begin{aligned} \rho _{+}^2\le C_\mathrm{mon}\rho _\delta ^2\le C_\mathrm{mon}\delta ^2=q_\mathrm{opt}\rho _\star ^2. \end{aligned}$$

*Step 3* Finally, the assumptions on the refinement strategy are used. The overlay estimate and Step 1 give

$$\begin{aligned} |\mathcal {K}_{+}|-|\mathcal {K}_\star |\le (|\mathcal {K}_\delta |+|\mathcal {K}_\star |-|\mathcal {K}_0|)-|\mathcal {K}_\star |=|\mathcal {K}_\delta |-|\mathcal {K}_0|\le { \Vert \phi \Vert _{\mathbb {A}_s}^{1/s} \delta ^{-1/s}.} \end{aligned}$$

Lemma 5.2 and (2.12) show

$$\begin{aligned} |\mathcal {R}_\star (\mathcal {T}_+)|\le C_\mathrm{rel}| [\mathcal {T}_\star ]{\setminus }[\mathcal {T}_{+}]|\le 2 C_\mathrm{rel}(|\mathcal {K}_{+}|-|\mathcal {K}_\star |). \end{aligned}$$

Combining the last two estimates, we end up with

$$\begin{aligned} |\mathcal {R}_\star (\mathcal {T}_+)|\le {2C_\mathrm{rel}}\Vert \phi \Vert _{\mathbb {A}_s}^{1/s}\alpha ^{-1/2s}\rho _\star ^{-1/s}, \end{aligned}$$

This proves (5.18) with $C_1={2C_\mathrm{rel}}$ and $C_2=\alpha ^{-1/2}$. By (5.20) we can apply Lemma 5.4 and see (5.19). $\square$

So far, we have only considered the auxiliary estimator $\rho _\star$. In particular, we did not use Algorithm 3.1, but only the refinement strategy $\mathtt{ref}(\cdot )$ itself. For the proof of optimal convergence (3.7), we proceed similarly as in [8, Theorem 8.4 (ii)].

### Proof of (3.7)

Due to (5.2), there is a constant $C\ge 1$ which depends only on $\check{\kappa }_{\max }$ with $\mu _\star ^2\le C\rho _\star ^2$ for all $[\mathcal {T}_\star ] \in [\mathbb {T}]$. We set $\theta _\mathrm{opt}:= {\overline{\theta }_\mathrm{opt}}/{C}$ and $\overline{\theta }:=C\,\theta$ and suppose that $\theta$ is sufficiently small, namely, $\theta <\theta _\mathrm{opt}$ and hence $\overline{\theta }<\overline{\theta }_\mathrm{opt}$. Let $\ell \in \mathbb {N}_0$ and $j\le \ell$. Choose a refinement $[\mathcal {T}_{+}]$ of $[\mathcal {T}_j]$ as in Lemma 5.6. In particular, the set $\mathcal {R}_j(\mathcal {T}_+)$ satisfies the Dörfler marking (5.20). According to (5.2), this implies

$$\begin{aligned} \theta \mu _j^2\le \overline{\theta }\rho _j^2\le \sum _{T\in \mathcal {R}_j(\mathcal {T}_+)}\rho _j(T)^2 \le \sum _{z \in \mathcal {N}_j \cap \bigcup \mathcal {R}_j(\mathcal {T}_+)} \mu _j(z)^2, \end{aligned}$$

i.e., the set $\mathcal {N}_j \cap \bigcup \mathcal {R}_j(\mathcal {T}_+)$ satisfies the Dörfler marking (3.3) from Algorithm 3.1. Since the chosen set $\mathcal M_j$ of Algorithm 3.1 has essentially minimal cardinality, we see with (5.18) that

$$\begin{aligned} |\mathcal M_j|\le C_\mathrm{mark}|\mathcal {N}_j\cap \bigcup \mathcal {R}_j(\mathcal {T}_+)|\le 2C_\mathrm{mark}|\mathcal {R}_j(\mathcal {T}_+)|\le 2 C_\mathrm{mark}C_1C_2^{1/s}\Vert \phi \Vert _{\mathbb {A}_s}^{1/s}\rho _j^{-1/s} \end{aligned}$$

With the mesh-closure estimate of (M3), we get

$$\begin{aligned} |\mathcal {K}_\ell |-|\mathcal {K}_0|\le C_\mathrm{mesh}\sum _{j=0}^{\ell -1} |\mathcal M_j|\le & {} 2C_\mathrm{mark}C_\mathrm{mesh}C_1C_2^{1/s} \Vert \phi \Vert _{\mathbb {A}_s}^{1/s} \sum _{j=0}^{\ell -1} \rho _j^{-1/s}\\\le & {} 2C_\mathrm{mark}C_\mathrm{mesh}C_1C_2^{1/s} C^{1/s}\Vert \phi \Vert _{\mathbb {A}_s}^{1/s} \sum _{j=0}^{\ell -1} \mu _j^{-1/s}. \end{aligned}$$

Linear convergence (3.5) shows

$$\begin{aligned} \mu _\ell \le C_\mathrm{lin}q_\mathrm{lin}^{\ell -j} \mu _j\quad \text {for all }j=0,\dots ,\ell . \end{aligned}$$

Hence,

$$\begin{aligned} |\mathcal {K}_\ell |-|\mathcal {K}_0|\le & {} 2C_\mathrm{mark}C_\mathrm{mesh}C_1C_2^{1/s} C^{1/s}\Vert \phi \Vert _{\mathbb {A}_s}^{1/s} \sum _{j=0}^{\ell -1} \mu _j^{-1/s}\\\le & {} 2C_\mathrm{mark}C_\mathrm{mesh}C_1(C_2C_\mathrm{lin}C)^{1/s} \Vert \phi \Vert _{\mathbb {A}_s}^{1/s}\mu _\ell ^{-1/s}\sum _{j=0}^{\ell -1} (q_\mathrm{lin}^{1/s})^{\ell -j}\\\le & {} (C_2C_\mathrm{lin}C)^{1/s} \frac{2C_\mathrm{mark}C_\mathrm{mesh}C_1}{1-q_\mathrm{lin}^{1/s}} \Vert \phi \Vert _{\mathbb {A}_s}^{1/s}\mu _\ell ^{-1/s}. \end{aligned}$$

This concludes the proof. $\square$

## Proof of Theorem 3.4, plain convergence (3.10)

To prove convergence of Algorithm 3.1 driven by the Faermann estimators $\eta _\ell$, we apply an abstract result of [23, Section 2] which is recalled in the following: Let ${\mathcal {H}}$ be a Hilbert space with dual space ${\mathcal {H}}^*$ and $V:{\mathcal {H}}\rightarrow {\mathcal {H}}^*$ be a linear elliptic operator and $f\in {\mathcal {H}}^*$. Let $(\mathcal {X}_\ell (f))_{\ell \in \mathbb {N}_0}$ be a sequence of finite dimensional nested subspaces of ${\mathcal {H}}$, i.e., $\mathcal {X}_\ell (f)\subseteq \mathcal {X}_{\ell +1}(f)$, with Galerkin approximations ${\varPhi }_\ell (f)\in \mathcal {X}_\ell (f)$ for the equation $V\phi =f$. Further, let $(\mathcal {N}_\ell (f))_{\ell \in \mathbb {N}_0}$ be a sequence of arbitrary finite sets and

$$\begin{aligned} \eta _\ell (f)&:=\eta _\ell (f,\mathcal {N}_\ell (f))\text { with }\eta _\ell (f,\mathcal {E}_\ell )\\&:=\left( \sum _{z\in \mathcal {E}_\ell } \eta _\ell (f,z)^2\right) ^{1/2}<\infty \quad \text {for all }\mathcal {E}_\ell \subseteq \mathcal {N}_\ell (f) \end{aligned}$$

some heuristical error estimator, where we only require $\eta _\ell (f,z)\ge 0$ for each $z\in \mathcal {N}_\ell (f)$. Let $(\mathcal M_\ell (f))_{\ell \in \mathbb {N}_0}$ be a sequence of marked elements with $\mathcal M_\ell (f)\subseteq \mathcal {N}_\ell (f)$ which satisfies the Dörfler marking, i.e.,

$$\begin{aligned} \theta \eta _\ell (f)^2\le \eta _\ell (f,\mathcal M_\ell (f))^2. \end{aligned}$$

Additionally let

$$\begin{aligned} {\widetilde{\rho }}_\ell (f):= & {} \widetilde{\rho }_\ell (f,\mathcal {N}_\ell (f))\quad \text {with }{\widetilde{\rho }}_\ell (f,\mathcal {E}_\ell )\\:= & {} \left( \sum _{z\in \mathcal {E}_\ell } {\widetilde{\rho }}_\ell (f,z)^2\right) ^{1/2}<\infty \quad \text {for all }\mathcal {E}_\ell \subseteq \mathcal {N}_\ell (f) \end{aligned}$$

be an auxiliary error estimator with local contributions ${\widetilde{\rho }}_\ell (f,z)\ge 0$. Then, there holds the following convergence result.

### Lemma 6.1

Suppose that $D\subseteq {\mathcal {H}}^*$ is a dense subset of ${\mathcal {H}}^*$ such that for all $f\in D$ and all $\ell \in \mathbb {N}_0$ there is a set $\mathcal {R}_\ell (f)\subseteq \mathcal M_\ell (f)$ such that the following assumptions (A1)–(A3) hold : 

1. $\eta _\ell (f)$ is a local lower bound of ${\widetilde{\rho }}_\ell (f){:}$ There is a constant $C_1>0$ such that
  $$\begin{aligned} \eta _\ell (f,\mathcal M_\ell (f))\le C_1 {\widetilde{\rho }}_\ell (f,\mathcal {R}_\ell (f))\quad \text {for all }\ell \in \mathbb {N}_0. \end{aligned}$$
2. ${\widetilde{\rho }}_\ell (f)$ is contractive on $\mathcal {R}_\ell (f){:}$ There is a constant $C_2$ such that for all $\ell \in \mathbb {N}_0, m\in \mathbb {N}$ and all $\delta >0,$ it holds
  $$\begin{aligned}&C_2^{-1}{\widetilde{\rho }}_\ell (f,\mathcal {R}_\ell (f))^2\le {\widetilde{\rho }}_\ell (f)^2-\frac{1}{1+\delta }\, {\widetilde{\rho }}_{\ell +m}(f)^2+(1+\delta ^{-1})C_2\Vert {\varPhi }_{\ell +m}(f)\\&\quad -{\varPhi }_\ell (f)\Vert _{{\mathcal {H}}}^2. \end{aligned}$$

In addition,  we suppose for all $f\in {\mathcal {H}}^*$ validity of : 

(A3) $\eta _\ell$ is stable on $\mathcal M_\ell (f)$ with respect to *f* :  there is a constant $C_3>0$ such that,  for all $\ell \in \mathbb {N}_0$ and $f'\in {\mathcal {H}}^*,$ it holds
  $$\begin{aligned} |\eta _\ell (f,\mathcal M_\ell (f))-\eta _\ell (f',\mathcal M_\ell (f))|\le C_3 \Vert f-f'\Vert _{{\mathcal {H}}^*}. \end{aligned}$$

Then,  there holds convergence $\lim _{\ell \rightarrow \infty }\eta _\ell =0$ for all $f\in {\mathcal {H}}^*$.

### Proof of plain convergence (3.10)

We choose ${\mathcal {H}}=\widetilde{H}^{-1/2}({\varGamma })$, ${\mathcal {H}}^*=H^{1/2}({\varGamma })$, *V* the weakly-singular integral operator (1.1). Moreover, Algorithm 3.1 generates the transformed NURBS spaces $\mathcal {X}_\ell (f)$, the set of nodes $\mathcal {N}_\ell (f)$, the Faermann estimator $\eta _\ell (f)$ and the set of marked nodes $\mathcal M_\ell (f)$. We use the mesh-size function $\widetilde{h}_\ell$ of Proposition 4.2 to define

6.1 $$\begin{aligned} {\widetilde{\rho }}_\ell (f,z):=\Vert \widetilde{h}_\ell ^{1/2}\partial _{\varGamma } (f-V{\varPhi }_\ell )\Vert _{L^2(\omega (z))}\quad \text {for all }z\in \mathcal {N}_\ell , \end{aligned}$$

if *f* is in the dense set $D=H^1({\varGamma })$. We aim to apply Lemma 6.1 and show in the following that (A1)–(A2) hold for all $f\in H^1({\varGamma })$ even with $\mathcal {R}_\ell (f)=\mathcal M_\ell (f)$ and that (A3) holds for all $f\in H^{1/2}({\varGamma })$. Then, Lemma 6.1 shows convergence (3.10) of the Faermann estimator.

1. Of Lemma 6.1 follows immediately from [24, Theorem 4.3], where the constant $C_1$ depends only on $\check{\kappa }_{\max }, p$, and $\gamma$.
2. Can be proved exactly as in [23, Section 2.4] as $\eta _\ell$ is efficient (see [25, Theorem 3.1]) and has a semi-norm structure. The constant $C_3$ depends only on ${\varGamma }$.

The only challenging part is the proof of (A2) for fixed $f\in H^1({\varGamma })$. We proceed similarly as in the proof of [23, Theorem 3.1]. In the following, we skip the dependence of *f*. The heart of the matter are the estimates $\widetilde{h}_{\ell +1}\le q_\mathrm{ctr}\widetilde{h}_{\ell }$ on $\omega _\ell (\mathcal M_\ell )$ and $\widetilde{h}_{\ell +1}\le \widetilde{h}_\ell$ on ${\varGamma }$, which follow from Proposition 4.2 and

$$\begin{aligned} \mathcal M_\ell \subset \bigcup ([\mathcal {T}_\ell ]{\setminus }[\mathcal {T}_{\ell +1}]). \end{aligned}$$

This shows

$$\begin{aligned} \widetilde{h}_\ell -\widetilde{h}_{\ell +m}\ge \widetilde{h}_\ell -\widetilde{h}_{\ell +1}\ge (1-q_\mathrm{ctr})\widetilde{h}_\ell \,\chi _{\omega _\ell (\mathcal M_\ell )}\quad \text {for all }\ell \in \mathbb {N}_0 \text { and }m\in \mathbb {N}. \end{aligned}$$

Hence, the estimator ${\widetilde{\rho }}_\ell$ satisfies

$$\begin{aligned} (1-q_\mathrm{ctr})\,{\widetilde{\rho }}_\ell (\mathcal M_\ell )^2/2\le & {} (1-q_\mathrm{ctr})\int _{\omega _\ell (\mathcal M_\ell )} \widetilde{h}_\ell \,|\partial _{\varGamma } (f-V{\varPhi }_\ell )|^2 dx\\\le & {} \int _{\varGamma } \widetilde{h}_\ell \,|\partial _{\varGamma }(f-V{\varPhi }_\ell )|^2-\int _{\varGamma }\widetilde{h}_{\ell +m} \,|\partial _{\varGamma }(f-V{\varPhi }_\ell )|^2 dx\\= & {} \Vert \widetilde{h}_\ell ^{\,1/2}\partial _{\varGamma }(f-V{\varPhi }_\ell )\Vert _{L^2({\varGamma })}^2-\Vert \widetilde{h}_{\ell +m}^{\,1/2}\partial _{\varGamma }(f-V{\varPhi }_{\ell })\Vert _{L^2({\varGamma })}^2. \end{aligned}$$

Here, the factor 1 / 2 on the left-hand side stems from the fact that each node patch consists (generically) of two elements. This fact also shows $\Vert \widetilde{h}_\ell ^{\,1/2}\partial _{\varGamma }(f-V{\varPhi }_\ell )\Vert _{L^2({\varGamma })}^2=\widetilde{\rho }_\ell ^{\,2}/2$. The Young inequality $(c+d)^2\le (1+\delta )c^2+(1+\delta ^{-1})d^2$ for $c,d\ge 0$, together with the triangle inequality shows

$$\begin{aligned} (1-q_\mathrm{ctr})\,{\widetilde{\rho }}_\ell (\mathcal M_\ell )^2/2\le & {} {\widetilde{\rho }}_\ell ^{\,2}/2 -\frac{1}{1+\delta }{\widetilde{\rho }}_{\ell +m}^{\,2}/2\\&+\,\frac{1+\delta ^{-1}}{1+\delta }\Vert \widetilde{h}_{\ell +m}^{1/2}\partial _{\varGamma } V({\varPhi }_\ell -{\varPhi }_{\ell +m})\Vert _{L^2({\varGamma })}^2. \end{aligned}$$

Finally, we use Proposition 4.1 and see

$$\begin{aligned} \Vert \widetilde{h}_{\ell +m}^{1/2}\partial _{\varGamma } V({\varPhi }_\ell -{\varPhi }_{\ell +m})\Vert _{L^2({\varGamma })}\le \widetilde{C}_\mathrm{inv} \Vert {\varPhi }_\ell -{\varPhi }_{\ell +m}\Vert _{\widetilde{H}^{-1/2}({\varGamma })}. \end{aligned}$$

with a constant $\widetilde{C}_\mathrm{inv}>0$ which depends only on $C_\mathrm{inv}$ and $h_\star \simeq \widetilde{h}_\star$. This yields

$$\begin{aligned} (1-q_\mathrm{ctr})\,{\widetilde{\rho }}_\ell (\mathcal M_\ell )^2/2\le {\widetilde{\rho }}_\ell ^{\,2}/2-\frac{1}{1+\delta }\,{\widetilde{\rho }}_{\ell +m}^{\,2}/2 +\frac{1+\delta ^{-1}}{1+\delta }\widetilde{C}_\mathrm{inv}^2\Vert {\varPhi }_\ell -{\varPhi }_{\ell +m}\Vert _{\widetilde{H}^{-1/2}({\varGamma })}^2. \end{aligned}$$

and concludes the proof of (A2) with $C_2=\max (\frac{1}{1-q_\mathrm{ctr}},2 C_\mathrm{inv}^2)$. $\square$
